# Multiple forms of protein–protein and DNA binding are exhibited by BrxC from the BREX phage restriction system

**DOI:** 10.1093/nar/gkag651

**Published:** 2026-07-02

**Authors:** Alexander J Kaiser, Jennifer J Readshaw, Lindsey A Doyle, Maria Puiu, Abigail Kelly, Sydney F McGuire, Julieta Peralta Acosta, Duc Vu, Andrew Nelson, Darren L Smith, Lidia Araújo-Bazán, Ernesto Arias-Palomo, Yvette A Luyten, Barry L Stoddard, Tim R Blower, Brett K Kaiser

**Affiliations:** Division of Basic Sciences, Fred Hutchinson Cancer Center, 1100 Fairview Ave. N., Seattle, WA 98109, United States; Department of Biosciences, Durham University, Stockton Road, Durham, DH1 3LE, United Kingdom; Division of Basic Sciences, Fred Hutchinson Cancer Center, 1100 Fairview Ave. N., Seattle, WA 98109, United States; Department of Biosciences, Durham University, Stockton Road, Durham, DH1 3LE, United Kingdom; Department of Biosciences, Durham University, Stockton Road, Durham, DH1 3LE, United Kingdom; Division of Basic Sciences, Fred Hutchinson Cancer Center, 1100 Fairview Ave. N., Seattle, WA 98109, United States; Department of Biology, Seattle University, 901 12th Ave., Seattle, WA 98122, United States; Division of Basic Sciences, Fred Hutchinson Cancer Center, 1100 Fairview Ave. N., Seattle, WA 98109, United States; The Lunenfeld-Tanenbaum Research Institute, Mount Sinai Hospital, Toronto, ON M5G1×5, Canada; Department of Biology, Seattle University, 901 12th Ave., Seattle, WA 98122, United States; Department of Applied Sciences, University of Northumbria, Newcastle upon Tyne, NE1 8ST, United Kingdom; Department of Applied Sciences, University of Northumbria, Newcastle upon Tyne, NE1 8ST, United Kingdom; Centro de Investigaciones Biológicas Margarita Salas, CSIC, Calle Ramiro de Maeztu 9, Madrid, 28040, Spain; Centro de Investigaciones Biológicas Margarita Salas, CSIC, Calle Ramiro de Maeztu 9, Madrid, 28040, Spain; New England Biolabs, 240 County Road, Ipswich, MA 01938, United States; Division of Basic Sciences, Fred Hutchinson Cancer Center, 1100 Fairview Ave. N., Seattle, WA 98109, United States; Department of Biosciences, Durham University, Stockton Road, Durham, DH1 3LE, United Kingdom; New England Biolabs, 240 County Road, Ipswich, MA 01938, United States; Department of Biology, Seattle University, 901 12th Ave., Seattle, WA 98122, United States

## Abstract

Bacteriophage exclusion (BREX) defense systems restrict phage infection via inhibition of phage DNA replication, while also modifying and protecting the bacterial genome. Type I BREX systems encode six conserved proteins, including a site-specific DNA methyltransferase. Host methylation requires a subset of BREX proteins, whereas phage restriction generally requires them all, suggesting that distinct but overlapping complexes mediate these activities. Full details of the mechanism and regulation of BREX remain to be understood. Here, we characterize the behavior and structures of the conserved BrxC AAA+ ATPase protein. BrxC forms multiple assemblages—various self-associating multimers, as well as a complex with BrxB-PglZ—that can be uncoupled via distinct point mutations, leading to differing effects on host methylation versus phage restriction. BrxC’s self-association, as well as its ability to bind DNA, is regulated by ATP binding and hydrolysis; BrxA and BrxB appear to also regulate those behaviors. These collective results suggest that BrxC may play a key role in controlling the two activities of BREX, with BrxB, BrxC, and PglZ forming a core complex, and the equilibrium among competing assemblies containing those proteins modulating the balance between idling and activated restrictive states.

## Graphical Abstract

**Graphical Abstract:**
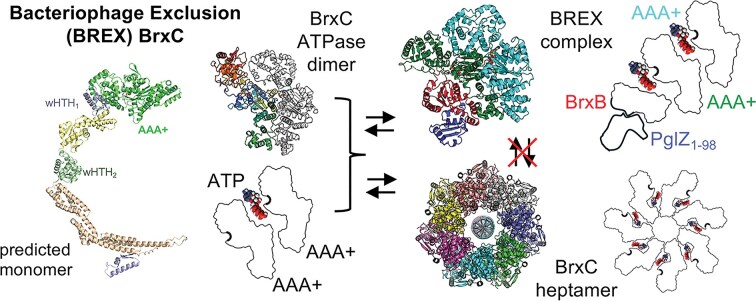

## Introduction

Antiviral defense in bacteria, commonly termed “phage restriction,” was first described in the early 1950s, when two groups of investigators characterized the ability of different strains of a given bacterial species to resist replication of phage during laboratory infection experiments [1, 2]. The subsequent identification of mechanisms, pathways, and factors that contribute to bacterial phage defense led to the identification of restriction endonucleases and their corresponding methyltransferase enzymes (i.e. “restriction-modification” or “RM” systems) [3–5], followed years later by the identification of CRISPR-based systems [6–10]. Both operate via site-specific cleavage of DNA targets within the invading phage genome, and thereby serve as innate and adaptive defense systems, respectively.

Many additional antiviral defense mechanisms and systems encoded in bacteria and archaea have also been identified and characterized, as summarized in [11]. Well over 100 such systems have been described; many were initially identified based on the location of their coding sequences within well-known genetic “defense islands” associated with mobile genetic elements [12]. These systems are notable for their diversity of form and function, ranging from those encoded by single genes and their corresponding products to highly complex multi-gene operons, collectively establishing multiple layers of antiviral defense [13]. Their diverse mechanisms of action extend beyond degrading phage DNA, and include detection of phage-specific proteins and effectors, synthesis of bacterial second messengers and antiviral factors, inhibition of phage genome replication, and/or triggering of bacterial cell stasis leading to cell death [11, 14].

One particularly complex group of phage restriction systems, found in roughly 10% of bacteria and archaea, is termed “Bacteriophage Exclusion” or “BREX” [15]. BREX systems operate by preventing phage DNA replication within the infected bacterial host cell, while also relying on the action of a site-specific methyltransferase to protect the bacterial host genome from its action. Originally discovered in the 1980s and first named “Pgl” based on its “Phage growth limitation” phenotype [16], BREX systems—currently classified into six types—comprise multiple genes (typically between 4 and 8) encoded within a single operon [15]. All BREX systems are built around a conserved core comprising BrxA, BrxB, BrxC, and PglZ (also termed BrxZ), and a sequence-specific DNA modifying enzyme (usually, but not always, a methyltransferase termed PglX or BrxX). In some cases, BrxA- and BrxB-like domains are embedded within other BREX polypeptides [17].

**Figure 1.**
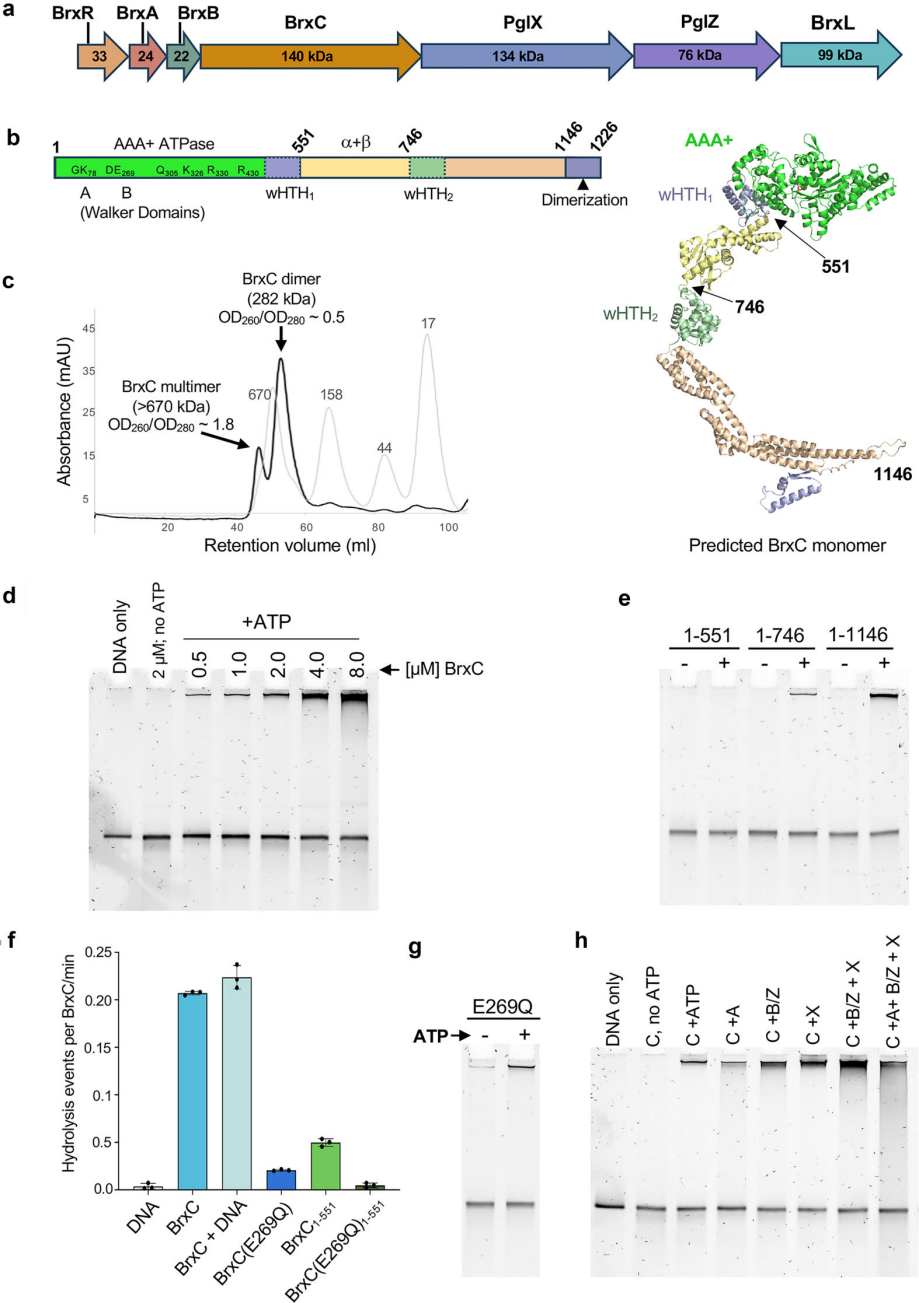
BrxC from *Acinetobacter* binds DNA in an ATP-dependent manner. (**a**) The BREX system from *Acinetobacter* (BREX^Aci^) comprises a single operon encoding seven proteins. (**b**) BrxC is a multidomain protein of ~140 kDa. The N-terminal region (residues 1–551) comprises an AAA+ ATPase domain with conserved Walker A and B motifs and basic residues that promote ATP-dependent multimerization, together with a winged helix-turn-helix (wHTH) domain. The remainder of the protein comprises a central region (residues 551–1146) predicted to adopt an extended architecture that includes an α + β domain (551–746), a second wHTH domain, and a C-terminal dimerization domain (residues 1146–1226). AF3 [33] prediction of BrxC^Aci^ (ipTM 0.98; pTM 0.59). The amino acid positions of truncations used in this study are indicated. (**c**) Size exclusion chromatographic analysis of full-length BrxC^Aci^, after an initial affinity tag purification step. BrxC^Aci^ elutes in two distinct peaks: an early-eluting peak associated with significant bound nucleic acid (OD260/280 ∼1.8), and a late-eluting peak corresponding to a BrxC^Aci^ dimer (∼280 kDa) with a much lower OD260/280 (∼0.5). The elution volumes of protein standard are indicated. (**d**) Electrophoretic mobility shift analyses (EMSA; gel shifts) of purified, DNA-free BrxC^Aci^ dimer shows ATP-dependent binding to double-stranded DNA (dsDNA). The indicated concentrations of BrxC were incubated with a 100 bp dsDNA in the absence of ATP (lane 2) or in the presence of 1 mM ATP. (**e**) EMSA assays with 2 µM final concentrations of the indicated BrxC^Aci^ truncations, incubated with and without ATP. (**f**) ATPase assays of the indicated BrxC^Aci^ constructs, each incubated at 1 µM concentration. Where indicated, 100 bp dsDNA was added at 50 nM final concentration. Triplicate data points are shown; error bars represent standard deviation. (**g**) EMSA assays of full-length BrxC^Aci^ and BrxC^Aci^(E269Q), each at a final concentration of 2 µM, in the presence and absence of ATP. (**h**) EMSA assays of full-length BrxC^Aci^ in the presence of the indicated BREX^Aci^ proteins, all at 2 µM final concentration. All reactions included ATP except where indicated for the second lane from the left.

“Type I” BREX systems rely on the action of up to seven distinct proteins (Fig. 1a), six of which have been individually characterized through structural, biochemical, and functional approaches. Those factors include (i) a sequence-specific transcriptional regulator termed BrxR—present in a subset of BREX systems and widely associated with diverse defense systems—that appears to regulate the activity of the system and maintain it in a repressed “stand-by” state prior to phage challenge [18–20]; (ii) the PglX sequence-specific DNA methyltransferase, which modifies a specific DNA target sequence throughout the host genome and thereby defines self versus non-self [21, 22]; (iii) PglZ, which exhibits nuclease activity; (iv) PglZ’s tightly associated binding partner BrxB, which contains an inactivated AAA+ ATPase domain [23]; (v) BrxA, a small DNA-binding protein of unknown recognition specificity [24]; and (vi) the DNA-binding AAA+ ATPase BrxL [25], which is replaced by various helicase enzymes in some non-Type I BREX systems [15].

The final BREX factor, BrxC, is the only BREX component for which no structural information has been reported. It is a large protein (>130 kDa) with an N-terminal AAA+ ATPase domain, a central region comprising two predicted wHTH domains that often function as DNA-binding motifs together with a coiled-coil domain, and a predicted C-terminal dimerization motif spanning the final 80 residues (Fig. 1b). Deletion of BrxC from an otherwise intact BREX system greatly reduces restriction activity; conversely, BrxC expression is strongly upregulated when the *Acinetobacter* BREX system is derepressed via deletion of BrxR—a response that may mimic the induction of BREX activity that occurs during phage infection [18]. Despite evidence suggesting a central role for BrxC in both the protective and restrictive activities of the BREX system, its behavior and role in either aspect of BREX function, such as perhaps modulating the switch from an inactive to active restriction system, have not yet been systematically examined.

The respective gene requirements for methylation or restriction have been characterized for several type I BREX systems. Across all systems examined, BrxB, BrxC, PglX, and PglZ are required for both methylation and restriction, whereas BrxA is required in some systems but not others [18, 21, 26–28]. BrxL is dispensable for methylation in all systems analyzed and displays variable importance in phage restriction [18, 21, 26–28]. Consistent with these gene requirements, pull-down analyses have detected complexes containing BrxB, BrxC, PglX, and PglZ proteins [21, 22], although stable assemblies of these factors have not been observed and remain uncharacterized. BrxB and PglZ form a particularly tight complex (“B:Z”) in widely diverged BREX systems [23]; given their conservation across BREX systems [17], a B:Z complex may serve as an “anchor” within larger BREX subcomplexes. The PglX methyltransferase recognizes and acts at six-basepair non-palindromic target sequences, but its activity requires BrxB, BrxC, and PglZ (and in some systems, BrxA) [18, 21, 26–28].

The mechanism of BREX phage restriction remains poorly understood and has been reported to not occur via wholesale degradation of the phage genome [26], as is observed in the presence of active restriction endonuclease or CRISPR nuclease systems. However, we recently showed that PglZ exhibits nuclease activity—detectable as both nicking and double-strand DNA cleavage [23]. Together, these data support a model in which overlapping sets of BREX proteins assemble into a methylation-directed complex under normal phage-free growth conditions and then reconfigure into a restriction-directed complex upon phage infection [21, 22, 26].

In this study, we characterize the solution behavior and multimerization properties of BrxC from a type I BREX system encoded in *Acinetobacter* and further demonstrate the generalizability and reproducibility of those results using a separate BREX system encoded in *Escherichia fergusonii*. We assess how specific residues and structural regions within BrxC (as well as ATP) drive those behaviors and describe a high-resolution cryogenic-election microscopy (cryo-EM) structure that defines the basis for its ATP-dependent self-association. We further demonstrate that BrxC can associate with dsDNA (and in doing so, forms higher-order multimers) and/or with the BrxB–PglZ complex. We then visualized the latter complex via additional X-ray crystallography and EM analyses, and demonstrate that single point mutations in either BrxC or in BrxB (which respectively abrogate BrxC self-association or BrxC association with BrxB), cause different effects on the ability of the *Acinetobacter* BREX system to carry out host methylation and/or phage restriction.

Based on our results, we propose that BrxC may play a central role in regulating BREX function, perhaps influencing the system’s switch from an idling restriction negative (“R-”) state in the absence of a phage challenge to a restriction active (“R+”) state when a phage challenge is sensed—e.g. through a change of its expression level, self-association, and/or binding affinities to other BREX factors and DNA.

## Methods

### Expression and purification of *Acinetobacter* BREX proteins

The constructs generated and used throughout this study are listed in Supplementary Table S1. Genes encoding the *Acinetobacter* BREX proteins were subcloned from the native operon into pET15b. BrxC^Aci^ and PglX^Aci^ were expressed with an N-terminal Twin-Strep tag containing a TEV protease cleavage site, while PglZ^Aci^ and BrxA^Aci^ were expressed with a C-terminal Twin-Strep tag containing a thrombin cleavage site. The gene encoding *Acinetobacter* BrxB was subcloned into pET24d+ with no affinity tag; BrxB^Aci^ was insoluble unless co-expressed alongside PglZ^Aci^. *Escherichia coli* BL21-CodonPlus (DE3)-RIL cells were transformed or co-transformed with the expression vectors and used for protein expression. For co-expression studies, pET15b and pET24d+ vectors containing various combinations of *Acinetobacter* BREX genes were co-transformed into *E. coli* and expressed as described below. In some cases, pairs of genes from the native *Acinetobacter* operon were subcloned into the same expression vector; examples include PglX^Aci^ and PglZ^Aci^, and BrxB^Aci^ and BrxC^Aci^. In co-expression experiments, only one gene contained a Twin-Strep tag for affinity purification.

The proteins were expressed using a previously described auto-induction protocol [29]. One liter of auto-induction media containing relevant antibiotics (0.1 mg/ml ampicillin for pET15b, 0.05 mg/ml kanamycin for pET24d) in 2.8 l flasks was inoculated with 10 single colonies and incubated for 9 h at 37°C, followed by 24 h at 18°C, shaking at 175 rpm. Cells were harvested by centrifugation at 7800 × *g* for 10 min at 4°C. Cell pellets were resuspended and washed in ice-cold Buffer W (200 mM NaCl, 100 mM Tris, pH 8.0, 1 mM ethylenediaminetetraacetic acid, pH 8.0), re-pelleted at 3200 × *g* for 12 min at 4°C, and stored at −20°C.

For IPTG (Isopropyl β-D-1-thiogalactopyranoside) inductions, one colony per 10 ml of media was used to inoculate LB (Luria-Bertani media) with antibiotics for overnight growth at 37°C with shaking at 175 rpm. The overnight cultures were diluted 1:100 to inoculate 1 l LB media containing relevant antibiotics in 2.8 l flasks. The outgrowths were incubated at 37°C, shaking at 175 rpm until an OD_600_ between 0.6 and 0.8 was reached; then, IPTG was added to a concentration of 0.5 mM, followed by 24 h at 18°C, shaking at 175 rpm. Cells were harvested by centrifugation at 7 800 × *g* for 10 min at 4°C. Cell pellets were harvested and stored as described earlier.

For purification, each cell pellet was resuspended in 25 ml Buffer W, followed by the addition Triton X-100 to a final concentration of 0.1%. Resuspended cells were lysed by sonication on ice and centrifuged in an SS34 rotor for 25 min at 18 000 × *g*, and the supernatant (soluble fraction) was filtered through 5 µm syringe filter. The filtered soluble fraction was then passed directly over 1 ml column volume (CV) Strep-Tactin-4Flow resin (IBA Life Sciences) via gravity filtration column (Bio-Rad), collected, and then passed over the column an additional time. The column and resin were then washed with 50 CVs of ice-cold Buffer W. Elutions were performed with six sequential additions of 0.75 CV Buffer E (IBA Life Sciences), each incubated for several minutes before collection. Biotinylated thrombin (EMD Millipore) was added to eluted protein (∼1 unit thrombin per mg of protein). For purifications containing BrxC or PglX, His6-tagged TEV protease was used, as thrombin had off-target activity with both proteins. The protease tag removal step was omitted from TST-tagged BrxC constructs lacking the TEV protease recognition motif. Removal of the TST tags was assessed via “pre-” and “post-protease” gels on 4%–12% BOLT sodium dodecyl sulfate–polyacrylamide gel electrophoresis (SDS–PAGE) gel in MES buffer (Invitrogen), while MOPS buffer (Invitrogen) was used with PglX and BrxC to achieve adequate separation for analysis. Samples were then concentrated to ∼2 ml in an Amicon Ultra centrifugal filter (10 000 and 30 000 MWCO; Millipore), filtered over a 0.22 µm spin filter, and loaded onto a HiLoad 16/600 Superdex 200 prep grade size exclusion column (Millipore Sigma) equilibrated in 25 mM Tris (pH 7.5), 200 mM NaCl. Peak fractions were pooled and concentrated to a final concentration (following addition of 10% glycerol) of 44 µM for all BREX proteins or constructs. Single-use aliquots were flash-frozen in liquid nitrogen and stored at −80°C.

Purification of co-expressed proteins was performed using the same procedure described earlier. In these experiments, one protein carried a Twin-Strep tag, while the co-expressed partner proteins were untagged.

### Expression and purification of *Escherichia fergusonii* BREX proteins

For large-scale expression of *E. fergusonii* BREX proteins, *E. coli* Rosetta (DE3) pLysS were transformed with pTRB791 (BrxC^Eferg^) and pTRB792 [BrxC^Eferg^(E268Q)]. *Escherichia coli* ArcticExpress (DE3) (Agilent) were transformed with pTRB789 (BrxC^Eferg^_1–551_) and pTRB790 [BrxC^Eferg^(E268Q)_1–551_]. All proteins were expressed with an N-terminal His_6_-SUMO tag containing a human sentrin/SUMO-specific protease 2 (hSENP2) cleavage site. BrxB and PglZ were expressed and purified as described previously [23].

For autoinductions, single colonies were used to inoculate 150 ml of LB with 0.1 mg/ml ampicillin and 0.025 mg/ml chloramphenicol for overnight growth at 37°C, shaking at 180 rpm. Starter cultures were re-seeded 1:100 (v/v) into 12 × 2 l baffled flasks containing 1 l ZY media and the relevant antibiotics and incubated at 37°C for 6 h, then at 18°C for 24 h, shaking at 150 rpm.

For IPTG inductions, single colonies were used to inoculate 70 ml of 2× YT with 0.1 mg/ml ampicillin and 0.01 mg/ml tetracycline for overnight growth at 37°C, shaking at 180 rpm. Starter cultures were re-seeded 1:100 (v/v) into 6 × 2 l baffled flasks containing 1 l 2× YT media and incubated at 37°C, shaking at 150 rpm until the OD_600_ reached ~0.6. The temperature was reduced to 12°C, IPTG was added to a final concentration of 1 mM, and expression was carried out for 48 h.

Cell pellets were harvested by centrifugation at 4000 × *g* for 30 min at 4°C. Cell pellets were resuspended in ice-cold A500 (500 mM NaCl, 20 mM Tris HCl, pH 7.9, 10 mM imidazole, 10% v/v glycerol). Resuspended pellets were disrupted by sonication on ice (45% amplitude, 10 s on, 20 s off pulse intervals, 3 min) and clarified by centrifugation at 48 000 × *g* for 1 h at 4°C. Clarified cell lysates were loaded onto a 5 ml HisTrap HP column (Cytiva) pre-equilibrated with A100 (100 mM NaCl, 20 mM Tris HCl, pH 7.9, 10 mM imidazole, 10% v/v glycerol). The HisTrap column was then washed with 50 ml A100.

For truncated BrxC proteins [BrxC^Eferg^_1–551_ and BrxC^Eferg^(E268Q)_1–551_], proteins were eluted directly onto a pre-equilibrated 5 ml HiTrap Q HP column using B100 (100 mM NaCl, 20 mM Tris HCl, pH 7.9, 250 mM imidazole, 10% v/v glycerol). The Q HP column was washed with 50 ml A100 and transferred to an Åkta^™^ Pure (Cytiva), and the target protein was eluted by anion exchange chromatography using a salt gradient from 100% A100 to 60% C1000 (1 M NaCl, 20 mM Tris HCl, pH 7.9, 10% v/v glycerol). Chromatographic peak fractions were collected, pooled, and incubated overnight in the presence of hSENP2 to facilitate the cleavage of the His-SUMO tag at 4°C. For full-length BrxC proteins [BrxC^Eferg^ and BrxC^Eferg^(E268Q)], proteins were eluted in B100 and dialysed overnight into 5 l A100 using Pur-A-Lyzer™ dialysis tubing with a MW cutoff of 6 kDa (Sigma–Aldrich) with gentle stirring at 4°C. The following day, the hSENP2-treated samples were applied to a second HisTrap HP column pre-equilibrated in A100. The flow-through containing untagged target protein was collected and concentrated by centrifugation using the appropriate MWCO Vivaspin concentrator (Sartorius). Concentrated truncated BrxC^Eferg^ protein samples were applied to a HiPrep^™^ 16/60 Sephacryl^®^ S-200 HR column (Cytiva), and full-length BrxC^Eferg^ protein samples were applied to a HiPrep^™^ 16/60 Sephacryl^®^ S-300 HR column (Cytiva), both pre-equilibrated with 1.2 CVs of sizing buffer (500 mM NaCl, 50 mM Tris HCl, pH 7.9, 10% v/v glycerol), for further purification by size exclusion chromatography (SEC). SEC peak fractions were pooled and analyzed by SDS–PAGE, then concentrated as described previously and quantified using a NanoDrop 2000 Spectrophotometer (Thermo Fisher). Final purified samples for biochemical analysis were resuspended in a 1:2 mixture of protein sample:storage buffer (500 mM NaCl, 50 mM Tris HCl, pH 7.9, 70% v/v glycerol) and flash-frozen in liquid nitrogen for storage at −80°C.

### Mass photometry

Mass photometry experiments were performed for both BREX systems on a Refeyn TwoMP instrument, using the AcquireMP 2024 R2 and DiscoverMP v2024 R2 for data acquisition and analysis, respectively. DiscoverMP v2024 R2 was used to create figures. For each sample to be tested, the droplet dilution autofocus function was used to find the focus plane using 18–19 µl of buffer (200 mM NaCl, 25 mM Tris) for *Acinetobacter* proteins, or 9 µl phosphate buffered saline for *E. fergusonii* proteins, on uncoated glass slides (Refeyn). One or two microliter of freshly prepared 100 nM protein sample was diluted directly into the drops (to reach final concentrations ranging from 5 to 10 nM), and 1 min video measurements were collected. *Escherichia fergusonii* proteins were pre-incubated in 1× ZB (150 mM NaCl, 50 mM Tris HCl, pH 8) with 10 µM MgCl_2_ in the presence and absence of 1 µM ATP for 1 h at 37°C. Experiments containing nucleic acids or protein:nucleic acid complexes were performed on glass slides (Refeyn) coated with poly-L-lysine (PLL). To prepare PLL-coated slides, 7 μl of 0.01% PLL (Sigma–Aldrich) was incubated between two coverslips for 30 s, then separated and immersed in MilliQ water to remove excess PLL. The treated surface was then rinsed directly with MiliQ water from a squirt bottle and dried with clean, pressurized air. MassFerence P1 Calibrant (Refeyn) standards were used according to manufacturer specifications. Gaussian fits were used for measurements unless otherwise specified.

### DNA binding

A 100 bp dsDNA containing a PglX^Aci^ recognition target sequence (5ʹ-GTAGAT-3ʹ; Supplementary Table S1) was used as the substrate in EMSA assays with BrxC^Aci^. The dsDNA template was ordered from Integrated DNA Technologies as a pre-annealed, HPLC-purified product. Twenty nanograms of DNA was used in binding reactions. Untagged BrxC^Aci^ protein was diluted in 150 mM NaCl, 25 mM Tris (pH 7.5), and used at final concentrations of 0.5–4 µM. Binding reactions (20 µl) were assembled in binding buffer (50 mM Tris, pH 7.5; 50 mM NaCl, 1 mM DTT, 5 mM MgCl_2_). When included, ATP was added at a final concentration of 1 mM. Reactions were incubated at 30°C for 2 h. Where indicated, 1 unit of proteinase K was added per 20 µl reaction and incubated for 30 min at 42°C. Samples were resolved on native 8% acrylamide gels (prepared using 29:1 acrylamide:bisacrylamide; Bio-Rad) in 0.5× TBE (Tris-borate-Tris-borate-ethylenediaminetetraacetic acid, or Tris-borate-EDTA) buffer. Five microliters of each binding reaction were loaded on the gel and run with 1× TBE running buffer at room temperature for 30 min at 150 V. Gels were stained for 30 min in SYBR Gold (Invitrogen) diluted in 1× TBE, rinsed twice with water, and imaged on a BioRad Gel Doc XR+ scanner.

### Protein–protein association pulldown assays

For pulldown assays using purified proteins from *Acinetobacter*, the protein isolated by affinity purification carried a Twin-Strep tag, whereas all partner proteins were either untagged or contained a His_6_-tag. The Twin-Strep-tagged protein was incubated at a two-fold molar excess (final concentration 2 µM) relative to the partner proteins (final concentration 1 µM) in binding buffer (150 mM NaCl, 25 mM Tris, pH 7.5) in 50 µl reaction volumes for 25 min at 22°C. Strep-Tactin agarose beads (IBA Lifesciences Inc., cat # 2-1250-002) were prepared by pipetting 12 µl of a 50% slurry into a 0.6 ml Eppendorf tube and centrifuged at 2000 × *g* for 1 min. The supernatant was removed by aspiration using a 25-gauge needle attached to a vacuum apparatus, and 100 µl of water was added. This was repeated more with binding buffer. Final supernatant was removed by aspiration, and 50 µl of binding buffer was added to resuspend the beads.

After the protein incubation reactions were complete, samples were added to the Strep-Tactin beads, gently tapped to resuspend, and incubated on a LabQuake rotisserie at 4°C for 45 min, with beads resuspended by tapping every 10 min. The beads were centrifuged as earlier, a sample of the supernatant was collected (“flow-through”), and the remaining supernatant was removed by aspiration. The beads were then washed twice with binding buffer using the same procedure described earlier to prepare the beads, transferred to a fresh tube, and washed two additional times. After the final wash and aspiration, 40 µl of Buffer E elution buffer (X) was added and incubated at 22°C for 15 min. Samples were centrifuged for 1 min at 5000 × *g*, and 10 µl of the supernatant (“elution”) was removed without disturbing the beads. An equal volume of 2× BOLT SDS sample buffer (containing 100 mM DTT) was added. Samples were boiled and resolved on 4%–12% BOLT gels (Invitrogen) using MES running buffer for 25 min at 200 V. Gels were visualized by Coomassie blue staining or silver staining.

Purification of co-expressed proteins was carried out using the same procedure described earlier for individually expressed proteins.

### Analytical size exclusion chromatography

Analytical SEC was performed on an Åkta™ Pure FPLC system (Cytiva). Protein samples were made up to 10 µM in A-SEC buffer (150 mM NaCl, 20 mM Tris HCl, pH 7.9) to a final volume of 100 µl and incubated either at room temperature [BrxC^Eferg^ and BrxC^Eferg^(E268Q)] or at 37°C [BrxC^Eferg^_1–551_ and BrxC^Eferg^(E268Q)_1–551_] for 1 h with and without 1 mM ATP and 10 mM MgCl_2_. Samples were loaded onto a 100 µl capillary loop using a 100 µl Hamilton syringe. The loop was washed with 500 µl nuclease-free water, followed by 500 µl A-SEC buffer, before and between each run. Samples were loaded onto a Superdex™ 200 increase 10/300 GL SEC column (Cytiva), pre-equilibrated with 1.2 CV A-SEC buffer, by running A-SEC buffer through the loop at 0.375 ml/min. Samples were resolved across 1.2 CV. In cases where the content of chromatogram peaks required verification by SDS–PAGE, 0.5 ml fractionation was performed in 96-well deep-plate blocks.

### ATPase activity assays

ATPase activity assays with *Acinetobacter* and *E. fergusonii* BREX proteins were performed with BIOMOL® Green (Enzo Life Sciences) in a 96-well plate format. A standard curve was generated from serial dilutions of free phosphate and used to calculate phosphate released from experimental samples, from which ATP hydrolysis events per BrxC per minute (reported as nmol Pi·nmol BrxC^−1^·min^−1^) were calculated after subtracting the ATP negative control background. Experimental wells containing the protein of interest were set up in 50 μl total volumes; A-SEC buffer was used for BrxC^Eferg^ assays, while a buffer containing 150 mM NaCl and 20 mM Tris (pH 7.5) was used for BrxC^Aci^ assays. For reactions with BrxC^Eferg^ and BrxC^Aci^ constructs alone, proteins were incubated at 500 nM, 1 μM, and 2 μM. For complexing assays, BrxC^Eferg^_1–551_ and BrxC^Eferg^(E268Q)_1–551_ were incubated at 1 μM with PglZ^Eferg^ and/or BrxB^Eferg^ at 1 and 0.5 μM. BrxC^Aci^ constructs (including truncations and point mutants) were incubated at 1 μM with PglZ^Aci^/BrxB^Aci^ at 1 μM. For assays containing DNA, a 100 bp dsDNA containing a PglX^Aci^ recognition sequence (Supplementary Table S1) was added to a concentration of 50 nM and incubated with proteins at 1 μM each. Reactions were carried out with and without 100 μM ATP and 10 mM MgCl_2_ for 1 h at 37°C. Negative control wells contained ATP or protein only. Positive control wells contained 1 U FastAP thermosensitive alkaline phosphatase (Thermo Fisher Scientific). Reactions were terminated by the addition of 100 μl BIOMOL Green reagent, and the color was allowed to develop for 15 min. Absorbance was measured in 96-well format at 620 nm. Readings were blanked using BIOMOL Green reagent and interpolated on a standard curve to give the amount of free phosphate produced. Values were averaged from three independent wells.

### Cryogenic-election microscopy analyses

Flow charts and summaries of data collection and processing methods and corresponding structural modeling, as described below, are shown in Supplementary Fig. S1 and Supplementary Table S2.

### BrxC^Aci^_E269Q_ negative stain screening

Negative stain grids were prepared by applying 3 μl of SEC-purified sample at a concentration of ~0.02 mg/ml to a plasma-cleaned Formvar/Carbon 400-mesh Copper grid (Ted Pella). The sample was allowed to absorb for 30 s before wicking excess solution with filter paper. The grid was quickly washed two times in 30 μl drops of water and once in a 30 μl drop of 2% uranyl formate (UF), followed by a final staining for 60 s with another 30 μl drop of 2% UF. The grids were air-dried for at least 1 h. Grids were screened on a Talos L120C transmission electron microscope (Thermo Fisher), operating at 120 kV and equipped with a 4k × 4k Ceta CMOS high-resolution 16M camera (Thermo Fisher). The sample distributed homogeneously and at random orientations over the surface of the prepared negative stained grids.

### BrxC^Aci^_E269Q_ data collection and processing (Supplementary Fig. S1a)

C-Flat 1.2/1.3 holey carbon film-coated 300-mesh copper grids (Electron Microscopy Sciences) were prepared for cryo-EM by plasma cleaning with water vapor using a Tergo EM Plasma Cleaner (Pie Scientific). Three microliters of SEC-purified sample at a concentration of 0.4 mg/ml was applied to the prepared grids, which were then blotted for 5.0 s at a tension of 0 and plunge-frozen into liquid ethane using a Mark IV Vitrobot (Thermo Fisher). A dataset of 4131 movies was collected at a super-resolution pixel size of 0.56 Å using a Glacios 200 kV electron microscope (Thermo Fisher) equipped with a Gatan K3 direct electron detector. Movies were imported into CryoSPARC [30], motion- and CTF-corrected, denoised using 100 training micrographs, and particles were picked by automated searching for Gaussian signals on 200 micrographs. This resulted in ~100 000 particles, which were extracted to a box size of 360 pixels (px) , Fourier-cropped to 180 px, and classified into 50 2D classes. Eleven classes containing ~40 000 particles with the best alignments were selected and passed into an Ab-initio 3D reconstruction job with four classes, followed by further Heterogenous Refinement (Hetero refinement), resulting in three classes containing the majority of particles and a single junk class containing three particles. The particles from the three non-junk classes were again sorted into 50 2D classes, and the best alignments were chosen as templates for picking on the entire dataset. Approximately 1.77 million particles were extracted into a box size of 360 px and fed into a Hetero refinement job using initial volumes (three unique, one junk) from the particle subset Hetero refinement, resulting in two unique volumes and two junk volumes.

The twp unique volumes (subsets A and B with 460k and 550K particles, respectively) were further individually refined using Non-Uniform Refinement (NU refinement) [31]. The A and B NU refinement volumes were individually subjugated to Hetero refinement, with input volumes for volume A being two copies of the NU refinement volume and for volume B being the NU refinement volume plus a junk volume. This resulted in two unique volumes from subset A and a third unique volume from subset B. All three unique volumes with their associated particles plus a junk volume were combined into a final Hetero refinement, resulting in three unique volumes, which were individually run through NU refinement. One volume required the hand to be flipped; then, the three volumes were aligned to allow for easier comparison. Two of the three volumes were run through Global and Local CTF refinement; the third volume did not benefit from these refinements. A final round of NU refinement was performed, resulting in three volumes with 477k, 315k, and 191k particles, and GSFSC resolutions of 2.95, 3.02, and 3.37 Å, respectively.

### Model fitting (Supplementary Fig. S1b)

Model fitting was primarily accomplished with the 2.95 Å volume. The volume was loaded into ChimeraX [32] along with a dimeric AlphaFold3 [33] model of BrxC^Aci^ extending from residues 1 to 1225 containing ATP. The model was roughly placed in the volume with the Fit in Map function, then refined with minor rebuilding using ISOLDE [34] and Coot [35]. Model fitting for the 3.02 and 3.37 Å volumes primarily used the model fit in the 2.95 Å volume and supplemented with the AlphaFold model for residues not present in the 2.95 Å resolution volume. Those models were also refined with minor rebuilding using ISOLDE and Coot.

### Visualization of *Acinetobacter* BrxC-B:PglZ complexes (Supplementary Fig. S1c–e)

The same general methods as BrxC^Aci^(E269Q) were followed for samples of a complex of the BrxC^Aci^ N-terminal domain (residues 1–553) bound to BrxB^Aci^ and PglZ^Aci^ with minor differences noted here. Stable particles containing all three subunits were generated by fusing the C-terminal end of BrxC^Aci^ to the N-terminal end of BrxB^Aci^ (a construct that was enabled by computational modeling using AlphaFold3 [33] in conjunction with crystallographic analyses described below), and then adding purified PglZ to that construct in a 1:1 molar ratio. Grids and corresponding data that led to visualization of a monomeric *Acinetobacter* C-B:Z complex contained no ATP or MgCl_2_ in the sample buffer. Grids and corresponding data that led to visualization of a dimeric *Acinetobacter* (C-B:Z)_2_ complex corresponded to a higher protein concentration (3.5 mg/ml) in the presence of 1 mM AMP-PNP, 1 mM MgCl_2_, and 0.05% CHAPS. Initial full model fitting was performed with AlphaFold3 models in ChimeraX using the “Fit in Map” tool. Individual domains were then further fit into volumes and minimally refined with ISOLDE to resolve distortions arising from manual fitting. Low resolution and lack of detail prevented further rebuilding and refinement.

### X-ray crystallography

Sequences encoding BrxC^Aci^ (E269Q)_1–551_ and PglZ^Aci^_1–98_ were subcloned into pET-based expression vectors containing protease-cleavable Twin-Strep affinity tags; wild-type (WT) BrxB^Aci^ was subcloned without affinity tags. WT BrxB^Aci^ and Strep-tagged PglZ^Aci^_1–98_ were co-expressed and co-purified, pulling down on PglZ^Aci^_1–98_, while BrxC^Aci^(E269Q)_1–551_ was expressed and purified separately. Affinity tags were removed by protease digest and further purified as described earlier prior to setting up crystallization trials. BrxC^Aci^(E269Q)_1–551_ and the PglZ^Aci^_1–98_:BrxB^Aci^ complex were incubated prior to crystallization for 30 min at room temperature in a 1:1 molar ratio in buffer with 1 mM ATP and 10 mM MgCl_2_ added. Crystals were grown by hanging-drop vapor diffusion in drops set with 1 µl of protein (protein concentration 9 mg/ml, 94 µM for each individual component) plus 1 µl of well solution (250 mM NaCl, 100 mM HEPES, pH 8.5, 21% PEG3350). For cryopreservation, cryoprotectant (300 mM NaCl, 100 mM HEPES, pH 8.5, 25% PEG3350, 25% 2-Methyl-2,4-pentanediol) was added to drops containing crystals through sequential additions and equilibrated for 1 min before flash freezing with liquid nitrogen.

The crystals were found to display I2 crystallographic symmetry, with unit cell dimensions *a* = 149.2 Å, *b* = 115.0 Å, *c* = 194.1 Å, and β = 108.0°. Data collection was conducted at beamline 5.0.1 the Advanced Light Source synchrotron beamline 5.0.1 at the Lawrence Berkeley National Laboratory in Berkeley, California. Data were collected on a Pilatus area detector and processing using program XDS. The X-ray diffraction data (Supplementary Table S3) were integrated and scaled to 2.74 Å resolution, corresponding to an I/σ(I) cutoff of 2.0. The structure was solved via molecular replacement (Top LLG = 2412; Top TFZ = 21), using the refined model of the BrxC^Aci^_1–551_ dimer solved by cryo-EM and an AlphaFold3 model of the BrxB^Aci^:PglZ^Aci^_1–98_ complex as sequential search models. MR produced a solution corresponding to an asymmetric unit containing two copies of a complex of *Acinetobacter* (BrxC_1–551_)_2_BrxB:PglZ_1–98_. The final values for R_work_/R_free_ were 0.195/0.250.

### Phage restriction assays

For assays testing the *Acinetobacter* BREX system, the obligately lytic bacteriophage λ_JL801_ was obtained from the Gerald Smith Lab at Fred Hutchinson Cancer Center. Serial dilutions of λ_JL801_ were prepared in phage buffer (50 mM Tris, pH 7.5, 25 mM NaCl, 4 mM MgSO_4_). *Escherichia coli* (NEB ER2683) was transformed with pACYC184 (“Empty vector”), pACYC BREX WT, pACYC BREX ΔBrxC, pACYC BREX ΔBrxB, pACYC BREX BrxC(E269Q), pACYC BREX BrxC(R330E), pACYC BREX BrxC(1–1146), pACYC BREX BrxC(1–551), or pACYC BREX BrxB(R82E), and grown overnight. Overnight cultures were diluted 1:33 into 20 ml of fresh LB containing 1.25 mM MgCl_2_ and grown to OD_600_ between 0.35 and 0.45. Two hundred microliters of the outgrowth cultures were pipetted into 4 ml of 0.5% molten LB agar containing 1.25 mM MgCl_2_ and poured onto 1.5% LB plates containing 1.25 mM MgCl_2_ and chloramphenicol (0.025 mg/ml). Once solidified, the plates were spotted with 5 µl 10-fold serial dilutions of λ_JL801_ and allowed to incubate overnight at 37°C before plaque-forming units (pfu/ml) were counted on each plate. Efficiency of plating (EOP) values were calculated by determining the phage titer on a test strain divided by the titer on the control strain.

For assays testing the *E. fergusonii* BREX system, *E. coli* DH5α were transformed with pTRB563 (pBrxXL), pTRB564 (pBrxXL-Δ*pglX*), pTRB565 (pBrxXL-Δ*brxU*), pTRB566 (pBrxXL-Δ*brxU*Δ*pglX)*, pTRB804 (pBrxXL-∆*brxC*), pTRB805 (pBrxXL-*brxC_1–551_*), pTRB806 (pBrxXL-*brxC(E268Q)*), a vector-only control (pTRB507), and grown overnight. Serial dilutions of phage Pau (from the Durham Collection [27]) were produced in phage buffer [10 mM Tris HCl, pH 7.4, 10 mM MgSO_4_, 0.01% (v/v) gelatin]. Two hundred microliters of overnight culture and 10 μl of phage dilution were added to a sterile 8 ml plastic bijoux with 3 ml of 0.35% (w/v) LB agar and poured onto LB plates. Plates were incubated overnight at 37°C before plaque forming units (pfu) were counted on each plate. EOP values were calculated by determining the phage titre on a test strain divided by the titre on a control strain.

For each construct tested, a minimum of three and up to five biological replicate experiments were performed, each of which corresponded to three technical replicates. Mean values and standard deviations were plotted in GraphPad Prism.

### Pacific Biosciences sequencing

Libraries for methylation sequencing were prepared using the SMRTbell HiFi 96 Prep kit (Pacific Biosciences). *Escherichia coli* strain ER2796 [36] was transformed with the respective constructs as for phage restriction assays above. Bacterial genomic DNA (gDNA) was extracted from 5 ml of overnight culture using a Monarch Genomic DNA Purification Kit (New England Biolabs #T3010). Bacterial gDNA samples were then sheared using a Qiagen Tissue Lyser II at 30 Hz for 240 s to produce DNA fragments with a mean size of 8–10 kb. The DNA was damage- and end-repaired, and SMRTbell adapters were then ligated. Exonuclease treatment removed non-incorporated SMRTbell DNA. Sequencing was performed on a PacBio Revio (Pacific Biosciences). Data were analyzed using PacBio SMRTAnalysis on SMRTLink_25.1 software Base Modification Analysis for Sequel data, to identify DNA modifications and their corresponding target motifs.

## Results

### BrxC ATP-dependent assembly and DNA-binding behavior

The results presented below primarily focus on BrxC and associated proteins from *Acinetobacter* (BrxC^Aci^), which are then supported by comparative experiments using BrxC from *E. fergusonii* (BrxC^Eferg^). The first step of this project was to purify the full-length BrxC^Aci^ and BrxC^Eferg^ homologs and assess their solution behaviors, self-assemblage states, and possible DNA-binding activities.

### BrxC purification and initial solution behavior

Full-length WT BrxC^Aci^ and BrxC^Eferg^ homologs were each expressed and purified from *E. coli* as described in “Methods” section. SEC of both constructs revealed two sequentially eluting species (Fig. 1c and Supplementary Fig. S2): an early-arriving peak corresponding to >670 kDa total mass (assessed relative to the largest molecular weight marker) with a high OD_260_/OD_280_ ratio >1.5, consistent with a large multimeric protein complex co-eluting with bound nucleic acid; and a later arriving, higher-amplitude, peak corresponding to a smaller complex with a mass of slightly <300 kDa and a much lower OD_260_/OD_280_ ratio (∼0.5–0.6), consistent with a BrxC dimer containing little or no bound nucleic acid. Once purified and separated from the larger protein–DNA fractions, the smaller species maintained the same elution behavior and apparent mass in subsequent SEC runs.

### BrxC DNA-binding and ATPase activity

The SEC profile described earlier led us to hypothesize that full-length BrxC purifies from *E. coli* as both a nucleic-acid-free dimer and a larger, heterogeneous population of DNA-bound multimers. The presence of a pair of wHTH domains in BrxC, which are commonly associated with DNA binding, further suggested that BrxC might interact with DNA. To test this possibility, we incubated the purified BrxC^Aci^ dimer with dsDNA (Supplementary Table S1) in the presence or absence of ATP and assessed binding by EMSAs. This analysis demonstrated ATP-dependent formation of a highly shifted BrxC^Aci^–DNA complex at low micromolar protein concentrations (Fig. 1d). Titration of BrxC^Aci^ in EMSA reactions induced a concentration-dependent increase in the highly shifted DNA species; however, complete shifting of the DNA substrate was not observed, even at the highest protein concentration tested.

To further characterize BrxC’s DNA-binding properties, we generated and purified truncated BrxC^Aci^ constructs corresponding to predicted boundaries between domains (indicated in Fig. 1b). These truncations included (i) residues 1–551 (BrxC^Aci^_1–551_), spanning the N-terminal AAA+ ATPase and the first wHTH domain; (ii) residues 1–746 (BrxC^Aci^_1–746_), which further incorporates the subsequent predicted central α + β domain; and (iii) residues 1–1146 (BrxC^Aci^_1–1146_), which includes all but the final 80 residues that are predicted to form a C-terminal protein dimerization interface. BrxC^Aci^_1–1146_ demonstrated DNA binding comparable to the full-length construct, indicating that the C-terminal dimerization domain does not significantly contribute to DNA binding. In contrast, BrxC^Aci^_1–746_ exhibited reduced but still detectable DNA binding, suggesting that the wHTH_2_ and/or second α + β domain contributes to DNA association. No binding was observed for BrxC^Aci^_1–551_, indicating that the α + β domain (amino acids 551–746) is also involved in DNA binding or priming wHTH_1_ for DNA interactions (Fig. 1e).

Given BrxC’s predicted N-terminal AAA+ ATPase domain, and wondering if such activity might influence DNA binding, we next measured the ATPase activity of both BrxC homologs. Full-length, WT BrxC^Aci^ and BrxC^Eferg^ each exhibited low but comparable levels of ATPase activity (Fig. 1f and Supplementary Fig. S3). Incubation with DNA induced only a marginal increase in BrxC^Aci^ (WT) activity. The activity of truncated BrxC^Aci^_1–551_ and BrxC^Eferg^_1–551_ constructs was substantially reduced compared to their full-length counterparts, despite the presence of an intact AAA+ ATPase domain (Fig. 1f and Supplementary Fig. S3). We also generated and tested mutants containing an E→Q point mutation in the conserved ATPase Walker B motif [BrxC^Aci^(E269Q) and BrxC^Eferg^(E268Q)]. Similar mutations in other AAA+ ATPases have been shown to still allow ATP binding while inhibiting ATP hydrolysis [37]. In both full-length constructs, this mutation caused significantly decreased ATPase activity (although some residual activity above background remained), and the same mutations in the 1–551 constructs further suppressed ATPase activity (Fig. 1f and Supplementary Fig. S3).

With these results in hand, we next asked whether reduced ATPase activity affected DNA binding. The full-length BrxC^Aci^(E269Q) mutant retained ATP-dependent DNA binding in EMSA assays (Fig. 1g). SEC analyses of BrxC^Eferg^(E268Q) similarly indicated dsDNA-binding activity, based on a similar elution profile as BrxC^Eferg^ WT (Supplementary Fig. S2b).

These experiments collectively indicate that (i) BrxC binds double-strand DNA in an ATP-dependent manner, (ii) ATP hydrolysis is not required for binding, and (iii) DNA binding appears to involve contributions from the α + β and wHTH domains.

### Effect of additional BREX factors on *Acinetobacter* BrxC DNA binding

We next assessed how other purified BREX proteins, individually and in combination, affected the interaction of BrxC^Aci^ with DNA. These included (i) BrxA^Aci^, (ii) a tightly associated complex of BrxB^Aci^ and PglZ^Aci^ (“B:Z”), and (iii) PglX^Aci^. Incubation of BrxC^Aci^ with equimolar amounts of B:Z or PglX^Aci^ individually did not alter DNA binding, and both factors induced a modest increase in the shifted band corresponding to a protein–DNA complex when titrated at higher molar ratios (Fig. 1h and Supplementary Fig. S4a). In contrast, BrxA^Aci^ reduced BrxC^Aci^ DNA binding. When B:Z and PglX^Aci^ were incubated together with BrxC^Aci^, an increase in the shifted protein–DNA complex was observed relative to either factor alone, whereas inclusion of BrxA^Aci^ with both factors reduced formation of the shifted band (Fig. 1h). In comparison to BrxC^Aci^, the B:Z complex and PglX induced only modest shifts when tested on their own, demonstrating the observed DNA binding behavior is driven by BrxC^Aci^ (Supplementary Fig. S4a).

Based on BrxA^Aci^’s ability to inhibit BrxC^Aci^ DNA binding in EMSAs, we tested whether the two proteins physically interact using a pull-down assay with purified proteins (Supplementary Fig. S4b). This analysis indicated a direct interaction between the two proteins. However, they did not co-elute on SEC, suggesting that the interaction may be low-affinity or require additional factors for stabilization. Together, the EMSA and protein interaction analyses may support a model in which BrxA modulates BrxC activity.

### BrxC self-association and multimerization

**Figure 2.**
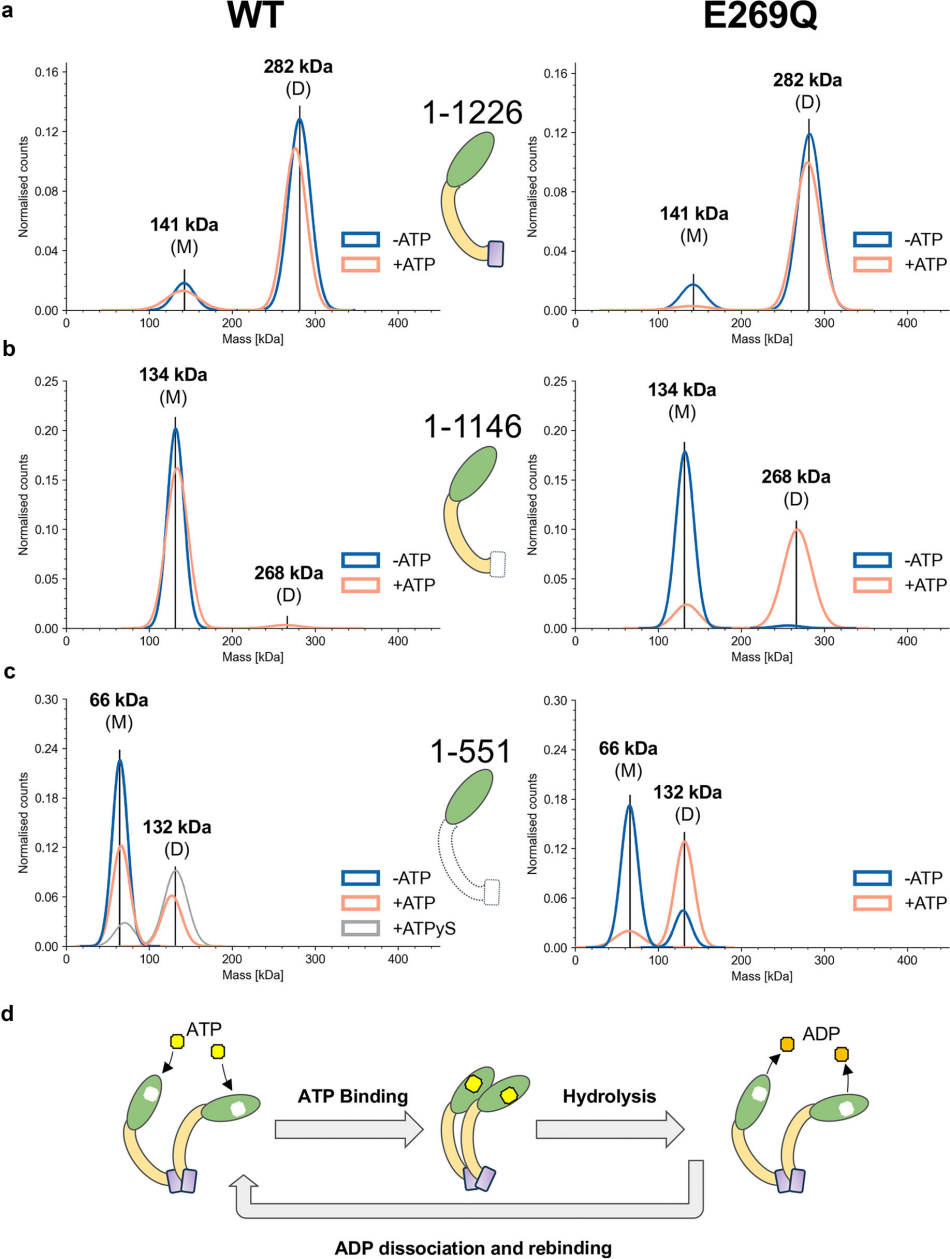
Solution behavior and ATP dependence of purified *Acinetobacter* BrxC constructs. Mass photometry was used to analyze the mass and multimerization behavior of full-length BrxC^Aci^ (BrxC^Aci^_1–1226_), BrxC^Aci^_1–1146_, and BrxC^Aci^_1–551_. Each construct was examined in the absence and presence of the inactivating E269Q point mutation in the ATPase Walker B motif (left plots and right plots, respectively) and in the absence and presence of ATP (purple and orange traces in each plot, respectively). (**a**) Full-length BrxC^Aci^ behaves largely as a dimer regardless of the presence or absence of ATP, both in its WT form (left) and with the E269Q mutation (right). (**b**) Deletion of the C-terminal region (residues 1147–1226) largely eliminates dimerization of the WT protein (left). Incorporation of the Walker B E269Q mutation rescues dimerization when ATP is present (right). (**c**) The N-terminal AAA+ domain (residues 1–551) is largely monomeric, with a measurable increase of the dimer in the presence of ATP (left). The fraction of ATP-dependent dimer is significantly increased by the Walker B E269Q mutation (right). (**d**) These analyses indicate that dimerization of BrxC is promoted both by its C-terminal region (residues 1147–1226) and by ATP-driven association of its N-terminal AAA+ ATPase domain. The latter association is further stabilized by the inactivating Walker B mutation (E269Q).

We next carried out a series of mass photometry experiments to further examine BrxC protein–protein association and dimerization behavior (Fig. 2). Three protein constructs were examined: full length BrxC^Aci^ (BrxC^Aci^_1–1226_), BrxC^Aci^_1–1146_ and BrxC^Aci^_1–551_. Each construct was analyzed in either the WT ATPase background or with an inactivating E269Q mutation in the Walker B motif (six constructs total), all in the presence or absence of ATP.

Full-length BrxC^Aci^ (WT) displayed a predominant mass of 282 kDa, corresponding to a protein dimer, and a minor monomeric species (141 kDa) regardless of the presence or absence of ATP (Fig. 2a, left). In contrast, the same full-length construct harboring the E269Q mutation displayed a nearly identical distribution in the absence of ATP, but in the presence of ATP was predominantly dimeric, with negligible monomeric BrxC^Aci^ observed (Fig. 2a, right).

Removal of the protein’s final 80 residues, generating BrxC^Aci^_1–1146_, resulted in a primarily monomeric species (134 kDa) in the presence or absence of ATP (Fig. 2b, left), indicating that the final 80 residues of the protein mediate dimerization. When the catalytic E→Q mutation in the Walker B motif was incorporated into this truncated construct (BrxC^Aci^(E269Q)_1–1146_), the protein remained monomeric in the absence of ATP; however, in the presence of ATP, a significant fraction shifted to 268 kDa, consistent with a dimeric species (Fig. 2b, right).

Finally, BrxC^Aci^_1–551_ was examined. In the absence of ATP, BrxC^Aci^_1–551_ (Fig. 2c, left) was entirely monomeric (66 kDa), whereas a small but measurable fraction dimerized in the presence of ATP. In contrast, for BrxC^Aci^(E269Q)_1–551_, a minor dimeric species was observed without ATP but shifted mostly to the dimeric state in the presence of ATP (Fig. 2c, right). We also tested the effect of the non-hydrolyzable analog ATPγS on BrxC^Aci^_1–551_ dimerization. In the presence of ATPγS, the majority of WT BrxC^Aci^_1–551_ was dimeric, phenocopying the E269Q mutant incubated with ATP (Fig. 2c, left). We did not observe higher order oligomers in these analyses.

Similar analyses with *E. fergusonii* BrxC constructs, utilizing analytical-SEC and mass photometry, largely reproduced the results described earlier (Supplementary Fig. S5); as with BrxC^Aci^, full-length BrxC^Eferg^ exists primarily as a dimer and the N-terminal ATPase domain in isolation (BrxC^Eferg^_1–551_) exhibits ATP-dependent dimerization. We observe low-level formation of complexes consistent with tetramers in full-length BrxC^Eferg^ constructs, though this assembly does not appear to be ATP-dependent. No higher-order complexes beyond dimers are observed for the BrxC^Eferg^_1–551_ constructs.

These results collectively suggest that BrxC contains two regions that independently promote dimerization: the protein’s N-terminal AAA+ ATPase domain, which undergoes reversible homodimerization that is regulated by ATP binding and hydrolysis, and its final ∼80 residues (residues 1147–1226), which forms a stable, ATP-independent dimerization interface. These observations suggest a model (Fig. 2d) in which BrxC dimerization is driven by two different regions of the protein and corresponding interactions: reversible ATP-binding and hydrolysis via the N-terminal ATPase domain, operating in concert with a constitutive dimerization motif located at the protein’s C-terminal end.

### A cryo-EM structure of the *Acinetobacter* BrxC dimer illustrates the involvement of ATP in protein–protein association

**Figure 3.**
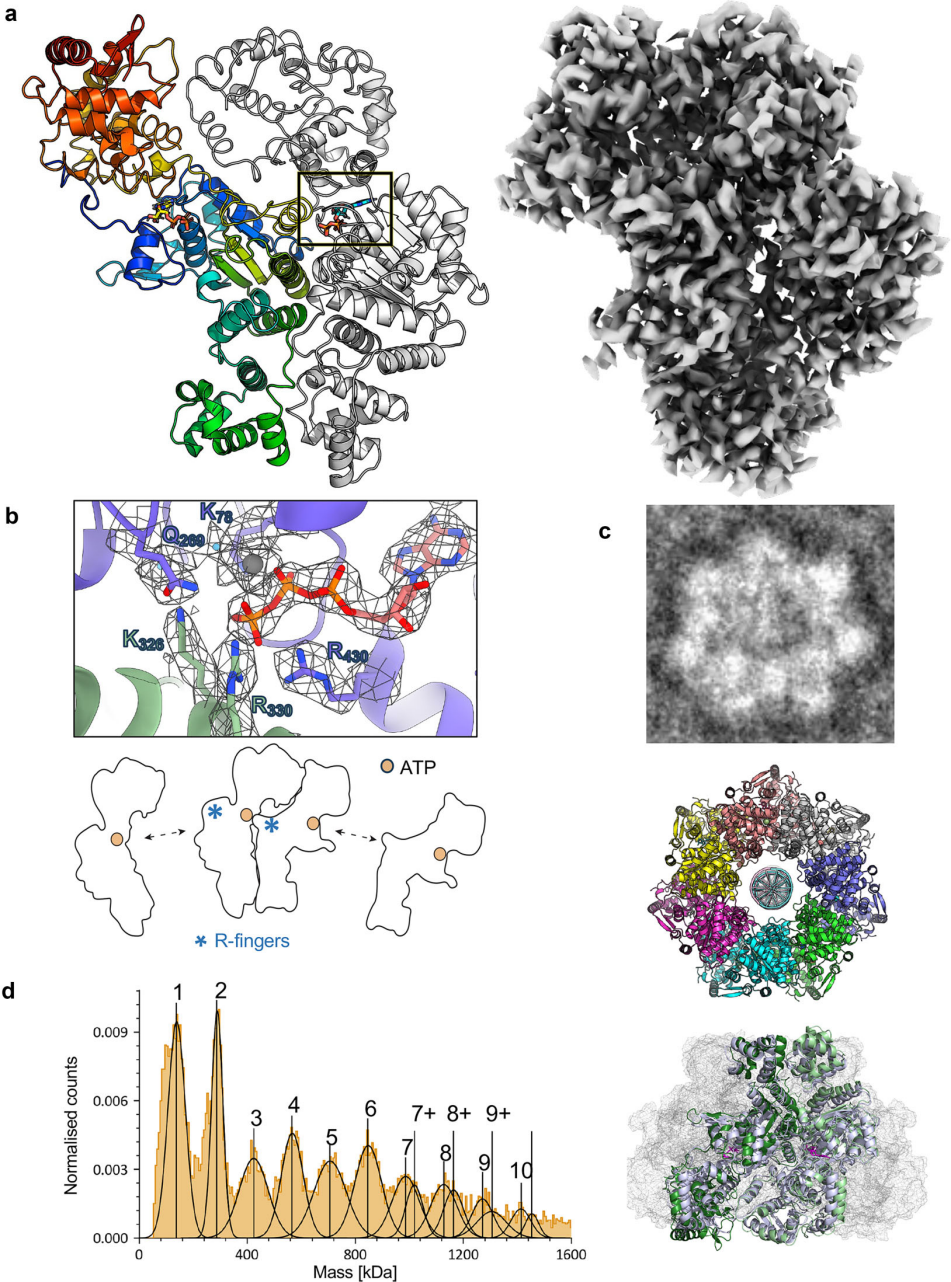
Cryo-EM analysis of the purified *Acinetobacter* BrxC dimer and DNA-dependent oligomerization. (**a**) Full-length, purified BrxC^Aci^(E269Q) was examined via negative stain and subsequent cryo-EM. The resulting map (right) provided clear detail, to an average of ∼2.9 Å resolution, for the N-terminal, ATP-bound domains (residues 1–551). At lower contour levels (Supplementary Fig. S1), additional density corresponding to BrxC^Aci^’s central region extending beyond the AAA+ domain was observable. In the cartoon image (left) one subunit is colored as a spectrum from its blue N-terminus to its red C-terminus; the second subunit is shown as a light gray ribbon outline. (**b**) Top: the indicated residues from each BrxC^Aci^ monomer interact with the bound ATP molecule and contribute to dimer formation. Bottom: BrxC^Aci^ monomers bind in a “back to face” packing arrangement to form a dimer, with Arg finger residues K326 and R330 from one subunit making contacts to the ATP bound in its partner subunit. Additional BrxC monomers could bind to BrxC dimers as depicted in the cartoon. (**c**) Top: Small numbers of particles observed throughout individual negative stain EM micrographs indicated the occasional presence of larger, well-ordered circular species displaying heptameric geometry. Middle: Predictive structural modeling of BrxC^Aci^ multimers using AlphaFold3 produced circular, closed heptameric rings with dimensions that closely matched the observed particles, with an interior pore of sufficient diameter to accommodate a DNA duplex (ipTM 0.55; pTM 0.60). Bottom: Overlay of cyro-EM BrxC^Aci^ dimer (green) onto the AlphaFold3-generated heptamer (gray). Two adjacent subunits of the heptamer are represented in cartoon format; the remaining five subunits are shown as wire mesh. Two ATP molecules are represented in magenta. Contact between amino acids interacting with ATP are conserved in both structures, while minor structural rearrangements are predicted away from the ATP-binding pocket. The dimeric structures appears compatible with higher order-oligomer formation. (**d**) Mass photometry of SEC-purified high-molecular weight fractions of BrxC ^Aci^ dimer incubated with 50 bp dsDNA. Numbers represent intervals of BrxC ^Aci^ monomer masses (140 kDa each), with the plus (+) sign corresponding to the additional mass of the dsDNA (33 kDa).

To determine the structural basis for the behaviors described earlier (Figs 1 and 2), we screened full-length BrxC^Aci^(E269Q) in the presence of ATP to produce uniform single-molecule particles suitable for visualization by cryogenic electron microscopy (cryo-EM). Our analysis (Supplementary Fig. S1a and b) revealed a dominant species corresponding to a dimer of BrxC^Aci^’s N-terminal domains. Electron density for this region was uniformly well resolved, with an average resolution of ∼2.9 Å. The structure showed the N-terminal domains arranged in an asymmetric “back-to-face” configuration, with ATP and coordinated magnesium ion positioned at the dimer interface and in the ATP binding pocket of the open-faced protomer (Fig. 3a). BrxC^Aci^ coordinates ATP using conserved AAA+ ATPase residues, including K78 (from the Walker A motif), E269 (Walker B motif), and R430 (Sensor 2 motif) from one BrxC^Aci^ subunit; and K326 and R330 (arginine fingers) from the partner subunit (Fig. 3b, top). The residues involved in ATP binding listed above are well conserved across multiple BrxC species, including those examined as part of this study (Supplementary Fig. S6) [37]. Analysis of this structure reveals a potential mode in which additional BrxC subunits could stack onto the BrxC dimer, mediated by interactions between the Arg finger residues of the incoming subunit and the ATP pocket of its binding partner (Fig. 3b, bottom).

Whereas the electron density surrounding BrxC^Aci^’s N-terminal domains was well defined, the remainder of the full-length protein was largely disordered, with the exception of much lower resolution density features extending part way into the protein’s central region (predicted to form mostly helical coiled-coil structural motifs and the associated wHTH_2_ domain) that were visible only at significantly lower contour levels (Supplementary Fig. S1a, bottom). It is likely that a combination of preferred orientation of the N-terminal domains on the grid and significant conformational heterogeneity extending beyond that region resulted in the lack of clear density for the remainder of the protein structure.

Close examination of negative stain grids containing SEC-purified high molecular-weight fractions of BrxC^Aci^ indicated the infrequent presence of higher-order circular protein assemblies with seven-fold (heptameric) symmetry (Fig. 3c, top). Similar heptameric ring-shaped particles were also observed for BrxC^Eferg^ by negative-stain TEM, albeit at low frequency within a heterogeneous background of protein species (Supplementary Fig. S7). The shape and dimensions of those particles correspond closely to an AlphaFold3 model in which a circular ring comprising seven BrxC^Aci^ subunits was predicted with high relative confidence (ipTM: 0.55, pTM 0.60) (Fig. 3c, middle; Supplementary Fig. S8). In that speculative model, as well as in the particles observed on negative stain EM images, a pore is present with an inner diameter sufficient to potentially accommodate a DNA duplex. To assess whether the BrxC dimer may be compatible with assembly into a heptamer complex, we superimposed the cryo-EM structure of the BrxC^Aci^ AAA+ dimers onto a pair of adjacent subunits in the AF3 heptameric model (Fig. 3c, bottom). The dimer aligns closely with this subunit pair (with an all atom RMSD of 3.1Å), suggesting that the observed dimeric architecture of BrxC is consistent with formation of a higher order heptameric state.

The lack of higher-order BrxC oligomer formation by the purified dimer (as observed in Fig. 2 mass photometry analyses), together with our prior observation of higher-order nucleic acid–associated protein complexes in SEC analyses (Fig. 1c) and the ability of dimeric BrxC^Aci^ to form high molecular weight DNA-bound complexes in EMSA assays (Fig. 1d, e, g, and h), suggested that DNA binding may promote BrxC oligomerization. To address this possibility, we incubated purified BrxC^Aci^ (E269Q) full-length dimer with a 50 bp dsDNA template and ATP, purified the resulting high-molecular-weight species by SEC, and analyzed the fraction by mass photometry (Fig. 3d). The fraction contained a stepwise distribution of oligomers corresponding to integer increments of BrxC^Aci^ monomers, and beginning at seven copies of BrxC^Aci^ and beyond, peaks corresponding to the additional mass of the 50 bp dsDNA are present. This analysis demonstrates that DNA promotes formation of BrxC oligomers, which includes heptameric and other higher-order assemblies, although the precise organization of these species and contribution of DNA to assembly remains to be determined.

### The BrxC N-terminal AAA+ domain also binds BrxB:PglZ

We previously observed that co-expression of *Acinetobacter* and *Salmonella* BrxB and PglZ resulted in stable binary B:Z complexes [19]. To determine whether larger *Acinetobacter* BREX assemblies could be detected, we carried out pull-down experiments using purified proteins or various co-expressed combinations of BREX constructs, with only a single construct affinity tagged in each experiment.

In our initial pull-down experiments, we observed the association of BrxB^Aci^, PglZ^Aci^, BrxC^Aci^(E269Q) and PglX^Aci^ (where PglZ^Aci^ contained a twin-strep affinity tag) when all four factors were co-expressed in *E. coli* (Supplemental Fig. S9), consistent with previous observations in other BREX systems [21, 22]. The identities of the co-eluting species in that experiment, including PglX^Aci^ and BrxC^Aci^ (which co-migrate by SDS–PAGE) were confirmed by mass spectroscopy. However, this complex did not remain stable on SEC, and we were unable to further structurally characterize the *Acinetobacter* B:C:X:Z complex.

**Figure 4.**
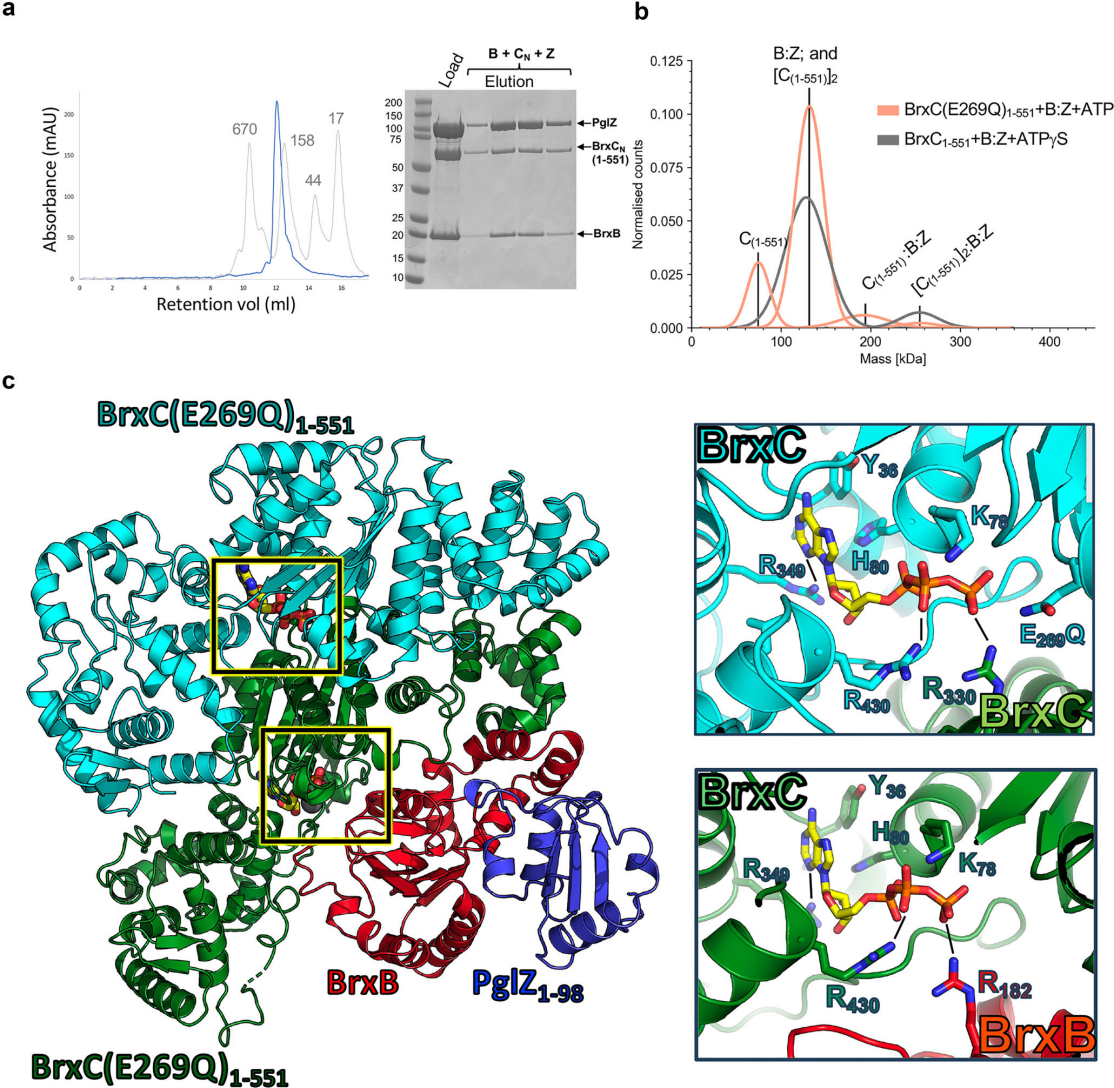
The N-terminal AAA+ domain of *Acinetobacter* BrxC binds the BrxB:PglZ subcomplex. (**a**) Co-expressed BrxB^Aci^, BrxC^Aci^_1–551_, and Strep-tagged PglZ^Aci^ were affinity purified by Strep-Tactin chromatography followed by SEC (left trace). All three proteins remain associated through this purification scheme (SDS–PAGE, right). (**b**) Two mass photometry experimental traces corresponding to BrxB^Aci^:PglZ^Aci^ heterodimer (“B:Z”) incubated with either [1] BrxC^Aci^_1–551_ and ATPγS (gray trace) or BrxC^Aci^ (E269Q)_1–551_ and ATP (orange trace). For each trace, masses are indicated on the *x*-axis and presumed molecular composition(s) are indicated above the peaks. The predicted mass of the B:Z complex is 122 kDa and that of the C_1–551_ monomer is 66 kDa. Complexes consistent with (C_1–551_)_1_:B:Z and (C_1–551_)_2_:B:Z stoichiometries are observed at 192 and 256 kDa, respectively. (**c**) X-ray crystallographic structure of a complex consisting of dimeric BrxC^Aci^(E269Q)_1–551_, full-length BrxB^Aci^ and PglZ^Aci^_1–98_. The BrxC-BrxC interface and the BrxB–BrxC interface (boxes and insets) involve comparable residues that bind a single copy of ATP between the corresponding protein subunits.

Subsequent co-expression experiments revealed a stable interaction between BrxC^Aci^’s AAA+ N-terminal domain (BrxC^Aci^_1–551_), BrxB^Aci^ and PglZ^Aci^. Co-expression of all three constructs in *E. coli*, with PglZ^Aci^ carrying a Twin Strep affinity tag, followed by affinity purification, proteolytic tag removal and SEC purification, resulted in co-elution of a stable complex containing all three proteins (Fig. 4a). Mass photometry analyses using purified proteins confirmed the ATP-dependent formation of complexes with stoichiometries (BrxC^Aci^_1–551_)_1_:BrxB^Aci^:PglZ^Aci^ (i.e. 1:1:1) and (BrxC^Aci^_1–551_)_2_:BrxB^Aci^:PglZ^Aci^ (i.e. 2:1:1, Fig. 4b), with dimerization of BrxC^Aci^_1–551_ promoted by ATPγS.

We next determined a high-resolution x-ray crystallographic structure of a stable ternary complex containing the interacting regions of BrxB^Aci^, BrxC^Aci^, and PglZ^Aci^. In our earlier cryo-EM work attempting to visualize B:Z complexes, we were unable to obtain high-resolution reconstructions, likely due to PglZ’s high flexibility from two predicted hinge regions and additional challenges resulting from the preferred orientation of the particles during EM analyses. To mitigate this issue, we purified and crystallized a minimal ternary complex that contained the N-terminal domain of PglZ^Aci^ (residues 1–98, which mediate its interaction with BrxB^Aci^ [23]), BrxC^Aci^(E269Q)_1–551_, and full-length BrxB^Aci^. The structure of the complex was solved using X-ray crystallography at a resolution of ∼2.7 Å (Supplementary Table S3 and Fig. 4c). The resulting structure consists of a dimer of BrxC^Aci^(E269Q)_1–551_ domains bound to a single copy of the BrxB^Aci^:PglZ^Aci^_1–98_ heterodimer (corresponding to a “(C_1–551_)_2_:B:Z_1–98_” stoichiometry).

In this complex, two bound ATP molecules and associated divalent cations are observed: one in the same position visualized previously between the two BrxC^Aci^ subunits (Fig. 4c, top inset), and a second in an equivalent position between one subunit of BrxC^Aci^ and BrxB^Aci^ (Fig. 4c, bottom inset). Whereas in the ATP-binding pocket formed between two BrxC^Aci^ subunits, a single arginine residue (R330) is contributed by one subunit, in the analogous pocket between BrxB^Aci^ and BrxC^Aci^, the functionally equivalent residue (R182) is instead contributed by BrxB^Aci^. A key insight from this structural result is that the presence of a bound BrxB^Aci^ subunit would preclude the binding of an additional BrxC^Aci^ subunit at that position.

**Figure 5.**
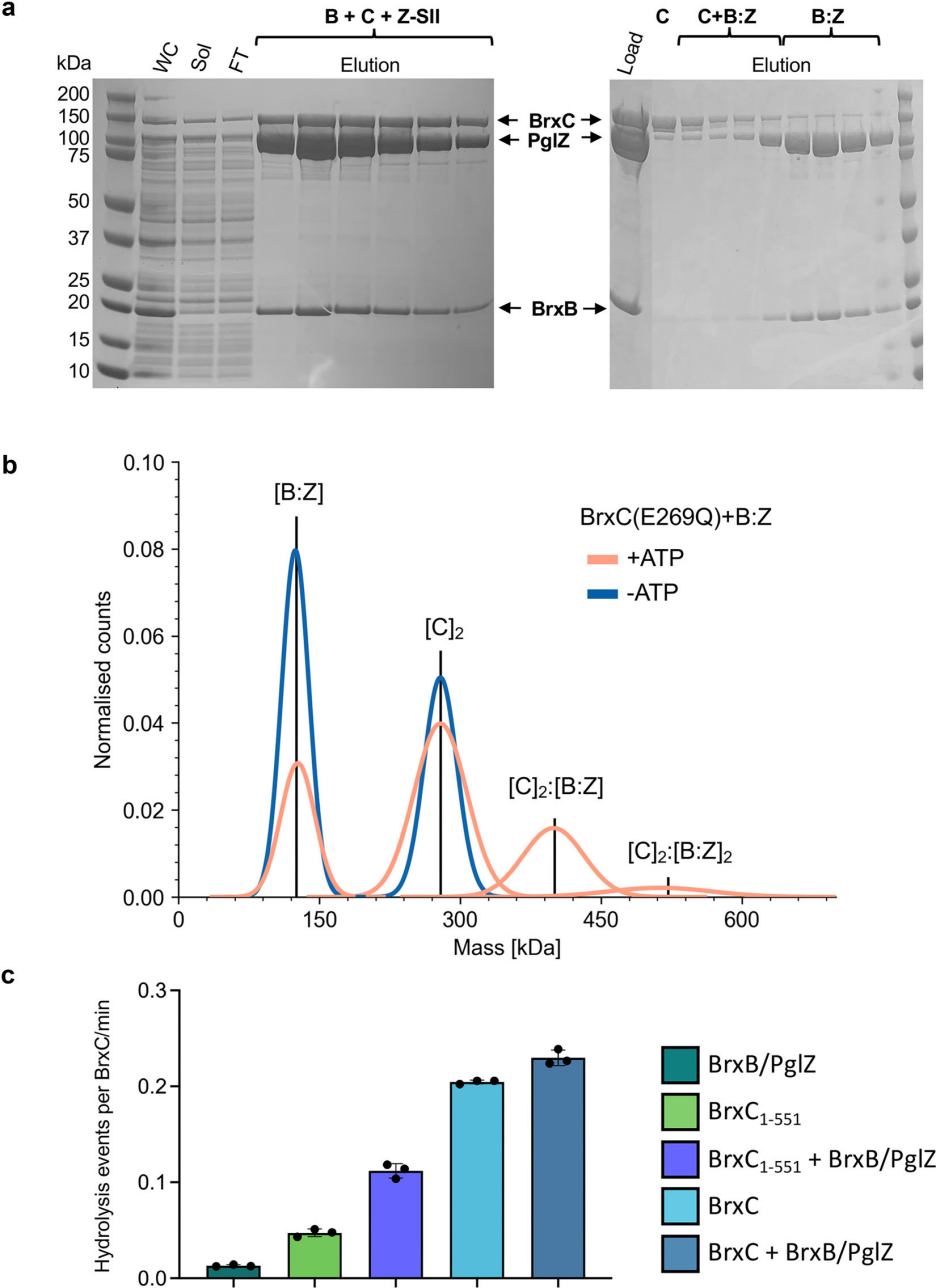
Formation of higher order stoichiometric complexes between full-length *Acinetobacter* BrxC and the BrxB:PglZ subcomplex. (**a**) Left: Co-expression of BrxB^Aci^, Strep-tagged PglZ^Aci^, and full-length BrxC^Aci^ demonstrates co-elution of all three subunits by Strep-Tactin affinity purification (WC: Whole cell lysate; Sol: filtered soluble lysate; FT: affinity column flow-through). (a) Right: The Strep-Tactin-eluted B:C:Z complex largely dissociates on SEC into BrxC^Aci^ dimers and the B^Aci^:Z^Aci^ complex. (**b**) Mass photometry analysis with full-length constructs of BrxC^Aci^ and co-purifed BrxB^Aci^:PglZ^Aci^. Complexes corresponding to C_2_:(B:Z) and C_2_:(B:Z)_2_ stoichiometries form in an ATP-dependent manner. (**c**) ATPase assays of BrxB^Aci^:PglZ^Aci^ and indicated BrxC^Aci^ constructs, comparing each protein alone, or incubated together at 1 µM concentration.

We then repeated the co-expression and purification scheme described earlier using full-length BrxC^Aci^ (rather than BrxC^Aci^_1–551_), along with full-length BrxB^Aci^ and full-length PglZ^Aci^ (Fig. 5). Similar to our prior result showing formation of stable complexes containing BrxC^Aci^_1–551_, BrxB^Aci^, and PglZ^Aci^, co-expression of BrxC^Aci^, BrxB^Aci^, and Strep-tagged PglZ^Aci^ resulted in co-elution of the three proteins from a Strep-Tactin affinity column (Fig. 5a, left). However, the resulting complex was less stable when purified by SEC, with BrxC^Aci^ largely dissociating from and eluting earlier than the tightly associated BrxB^Aci^:PglZ^Aci^ complex (Fig. 5a, right).

We further analyzed the association between purified full-length BrxC^Aci^, BrxB^Aci^, and PglZ^Aci^ constructs by mass photometry (Fig. 5b) and observed that the full-length proteins assemble into a mixture of C_2_:(B:Z)_1_ and C_2_:(B:Z)_2_ complexes (i.e. 2:1:1 and 2:2:2 stoichiometric ratios of C, B, and Z) in an ATP-dependent manner. The observed 2:2:2 complex demonstrates that both of BrxC’s AAA+ domains are accessible for DNA binding by the B:Z complex in the context of dimeric full-length BrxC.

We attempted to visualize Cryo-EM structures of these larger complexes of BrxC bound to the BrxB and PglZ by generating particles that contained either (i) all three separate protein chains or (ii) a pair of protein chains, with one corresponding to a fusion of BrxC^Aci^_1–551_ to BrxB and the second corresponding to PglZ (the second construct was motivated by our crystal structure, in which we observed that BrxC residue 551 is in proximity of the N-terminus of BrxB).

The latter strategy ultimately produced a low resolution (∼7 Å) density map (Supplementary Fig. S10) that could be readily superimposed against a model containing two copies of a complex of BrxC-B_fusion_:Z that was produced by AlphaFold3 (single-particle confidence: pTM = 0.54, ipTM = 0.7). In the map-docked model, dimerization is implied to be mediated by two regions of PglZ: (i) interactions between the central regions of each PglZ partner, and (ii) interactions between PglZ’s Ig-like C-terminal domains. This architecture may provide insight into the organization of higher-order (C_1–551_)_2_:B:Z complexes observed by mass photometry (Fig. 4b), although it remains unclear whether BrxC’s C-terminal domains dimerize in the full-length complex or perhaps exist as a mix of dimerized and un-dimerized states.

To investigate if the interactions between BrxC^Aci^, BrxB^Aci^, and PglZ^Aci^ described earlier are generalizable across homologous BREX systems, we further examined whether similar complexes form between individually purified protein constructs from *E. fergusonii* (Supplementary Fig. S11). Both BrxC^Eferg^_1–551_ and BrxC^Eferg^(E268Q)_1–551_ incubated with a mixture of BrxB^Eferg^ and PglZ^Eferg^ produced two early-eluting peaks by SEC (Supplementary Fig. S11a and c). Analysis of the early peak fractions by SDS–PAGE shows that in the absence of ATP, BrxC_1–551_ elutes at low levels alongside BrxB^Eferg^ and PglZ^Eferg^ (Supplementary Fig. S11b), and the presence of ATP and MgCl_2_ abolishes this interaction (Supplementary Fig. S11b, bottom). Comparatively higher amounts of BrxC^Eferg^(E268Q)_1–551_ were observed alongside BrxB^Eferg^ and PglZ^Eferg^ within the early eluting peak fractions both in the absence (Supplementary Fig. S11d, top) and presence (Supplementary Fig. S11d, bottom) of ATP and MgCl_2_. These data further support a model where (i) the AAA+ domain of BrxC forms a complex with BrxB and PglZ, (ii) the interaction is dependent upon or stabilized by the binding, but not hydrolysis, of ATP, and (iii) the interaction is conserved between homologous BREX systems found in different bacteria.

Having identified an ATP-dependent interaction between BrxC’s AAA+ domain, BrxB, and PglZ, we sought to analyze the effect, if any, that the interaction has on the ATPase activity of BrxC (Fig. 5c and Supplementary Fig. S12). We first tested the effect of BrxB^Aci^:PglZ^Aci^ on full-length BrxC^Aci^ and observed little to no change in ATPase activity. Because ATPase activity in the full-length protein may be influenced by dimerization of BrxC’s AAA+ domains, we tested the BrxC_1–551_ constructs from both species to better isolate the contribution of the B:Z complex. Under these conditions the B:Z complex induced a ∼2–6 fold increase in ATPase activity.

To determine the individual contributions of BrxB and PglZ to the observed increase in BrxC_1–551_ ATPase activity, we tested both *E. fergusonii* factors alone (Supplementary Fig. S12; BrxB^Aci^ could not be tested because it is only stable when co-purified with PglZ^Aci^). Incubation with BrxB^Eferg^ alone induced a ∼4-fold increase in ATPase activity of BrxC^Eferg^_1–551_ compared to each individual component, whereas the observed activity of BrxC^Eferg^_1–551_ incubated with PglZ^Eferg^ was comparable to the additive contribution of the individual components rather than an increase. This effect was further amplified by the BrxB^Eferg^:PglZ^Eferg^ complex, reaching a ∼6-fold increase in ATPase activity. BrxB^Eferg^ and BrxB^Eferg^:PglZ^Eferg^ also induced a small increase in the ATPase activity of BrxC^Eferg^(E268Q)_1–551_. These results indicate that the B:Z complex can regulate BrxC ATPase activity.

### Characterization of mutants that individually disrupt BrxC:BrxC or BrxB:BrxC association

**Figure 6.**
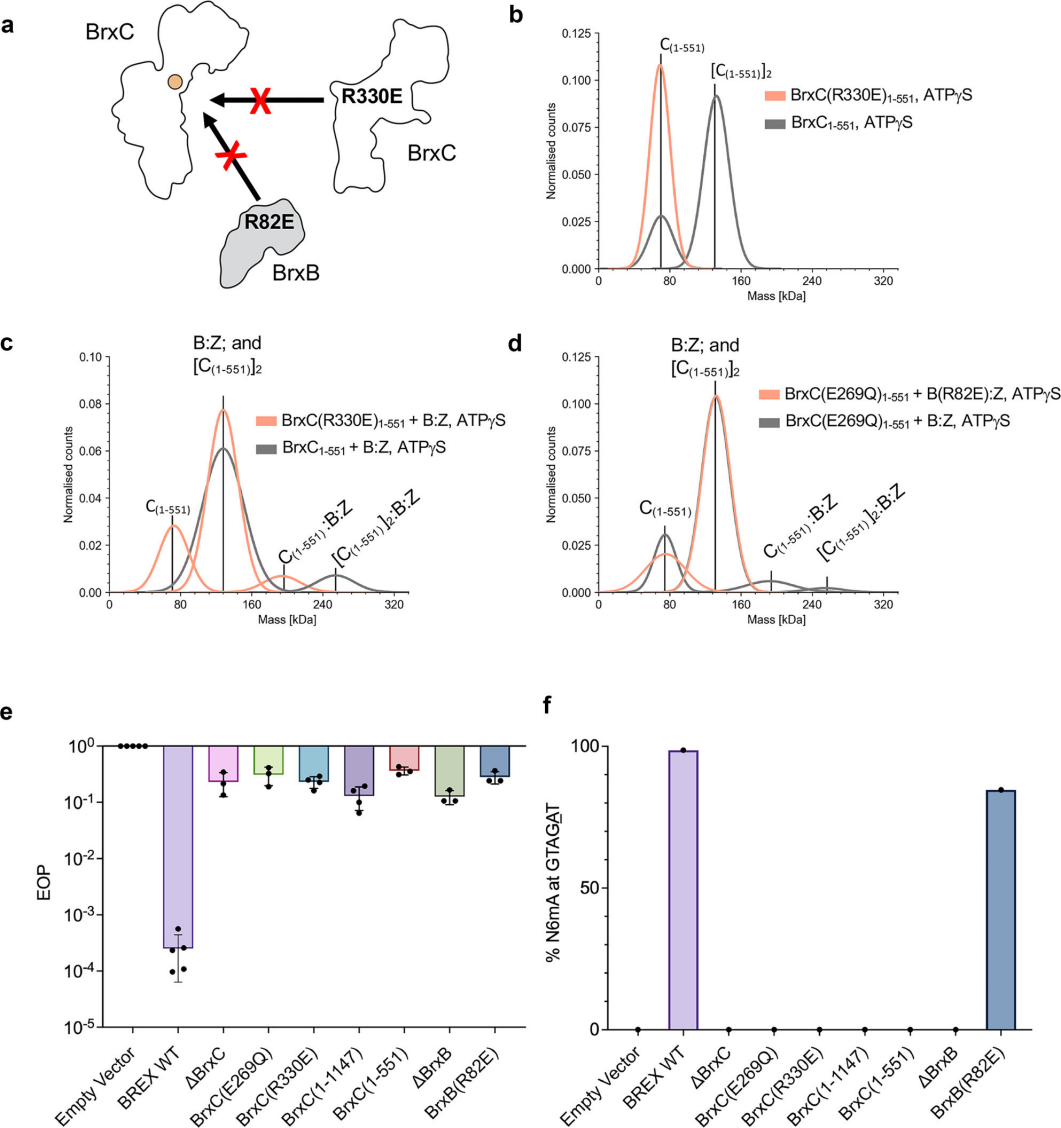
Disruption of BrxC:BrxC and BrxB:BrxC interfaces, and impact on *Acinetobacter* BREX activity. (**a**) Schematic depicting the BrxC^Aci^(R330E) and BrxB^Aci^(R82E) mutants predicted to disrupt the BrxC:BrxC and BrxB:BrxC interfaces, respectively. (**b**) Mass photometry analysis of WT BrxC^Aci^_1–551_ (gray) and BrxC^Aci^(R330E)_1–551_ (orange) in the presence of ATPγS shows that the R330E mutant blocks BrxC^Aci^ N-terminal self-association. (**c**) The B^Aci^:Z^Aci^ complex was incubated with BrxC^Aci^_1–551_ (gray trace) or BrxC^Aci^(R330E)_1–551_ (orange trace) in the presence of ATPγS. BrxC^Aci^(R330E)_1–551_ does not disrupt the BrxB–BrxC interaction but only forms a complex with B:Z in a (C_1–551_)_1_ B:Zstoichiometry (i.e 1:1:1). (**d**) BrxC^Aci^(E269Q)_1–551_ was incubated with BrxB^Aci^:PglZ^Aci^ or BrxB^Aci^(R82E):PglZ^Aci^ in the presence of ATPγS. This analysis shows that BrxB^Aci^(R82E) abrogates complex formation between B:Z and C_1–551_. (**e**) EOP results for lambda phage challenges against *E. coli* transformed with the indicated *Acinetobacter* BREX constructs and empty vector control. Error bars represent standard deviation from the mean of biological replicates. The truncations of BrxC^Aci^ and mutations disrupting the C–C or B–C interfaces are comparable to deletion of either gene individually and result in nearly complete loss of BREX-mediated protection. (**f**) PacBio genome methylation data of the same BREX constructs tested by EOP in panel (e). Sequencing percentage corresponds to the amount of methylated GTAGAT N6mA BREX motifs.

Given that BrxB associates with BrxC through the same interface that mediates higher-order BrxC multimerization—suggesting that these two binding events are mutually exclusive—we identified a mutation in each subunit designed to selectively disrupt one interaction without affecting the other. In the BrxB^Aci^–BrxC^Aci^ (“B–C”) interface, BrxB^Aci^ residue R82 forms a well-ordered electrostatic contact to D204 in BrxC^Aci^ (Fig. 6a). Conversely, in the BrxC^Aci^–BrxC^Aci^ (“C–C”) interface, R330 in one subunit interacts with bound ATP and with E289 in the other subunit but is not involved in the B–C interface.

We therefore purified variants carrying individual mutations [BrxB^Aci^(R82E) and BrxC^Aci^(R330E)_1–551_] and examined their ability to bind the corresponding WT counterparts using mass photometry. We used the truncated BrxC^Aci^_1–551_ construct for this analysis to uncouple dimerization of the N-terminal AAA+ domain from dimerization mediated by the C-terminal domain present in the full-length protein. As predicted, both charge reversal mutations selectively abrogated their corresponding interactions: BrxC^Aci^(R330E)_1–551_ selectively eliminated C–C self-association (Fig. 6b) but retained interaction with B^Aci^:Z^Aci^, albeit with only one copy of BrxC^Aci^_1–551_ (forming a BrxC_1–551_:B:Z complex with 1:1:1 stoichiometry) (Fig. 6c). BrxB^Aci^(R82E) abolished the B–C interaction (Fig. 6d).

We also tested how these individual mutants affected BrxC^Aci^_1–551_ ATPase activity (Supplementary Fig. S12). BrxC^Aci^(R330E)_1–551_ exhibited ATPase activity comparable to BrxC^Aci^_1–551_ alone and showed a similar increase in ATPase activity when incubated with BrxB^Aci^:PglZ^Aci^, consistent with the BrxC(R330E) mutant not affecting the B–C interface. In contrast, BrxB^Aci^(R82E):PglZ^Aci^ failed to stimulate BrxC^Aci^(WT)_1–551_ ATPase activity, consistent with this mutant disrupting the B–C interface.

### Impact of BREX mutations on phage restriction and methylation

Finally, we measured the impact of our studied mutations on the two biological outcomes of BREX activity, (i) phage restriction, and (ii) methylation of the genomic PglX^Aci^ target site (GTAGAT) when expressed in *E. coli*. Whereas BREX^Aci^ WT conferred 3–4 orders of protection against lambda phage challenge relative to an empty vector control, all mutants lost the ability to restrict lambda (Fig. 6e). In the corresponding methylation analysis, BREX^Aci^ WT conferred nearly complete methylation of PglX^Aci^ sites in the *E. coli* ER2796 [36] genome (98.6%) (Fig. 6f). All constructs except the BrxB^Aci^(R82E) mutant demonstrated complete loss of methylation activity; the BrxB (R82E) mutant conferred 84.6% coverage (Fig. 6f). Although slightly reduced relative to BREX^Aci^ WT, BrxB^Aci^(R82E) mutant displays an uncoupling of phage protection and methylation previously observed for point mutations in *Salmonella* BrxC’s C-terminal domain [38].

Corresponding analysis with BREX^Eferg^ produced a different result; instead of being inactivated, phage restriction was still observed for BrxC^Eferg^(E268Q) and an intermediate restriction phenotype was observed for BrxC^Eferg^_1–551_ (Supplementary Fig. S13a). It was noted that transformation efficiency to produce both these mutant strains was very low, and the cells grew slowly. Interestingly, methylation analysis of both mutations showed no BREX methylation activity (Supplementary Fig. S13b). Lack of methylation would likely account for the slow growth of these strains if BREX remained sufficiently active to protect against phages while also being modestly toxic to the unmethylated host. This result reflects another nuance in BREX activity between homologous systems.

## Discussion

**Figure 7.**
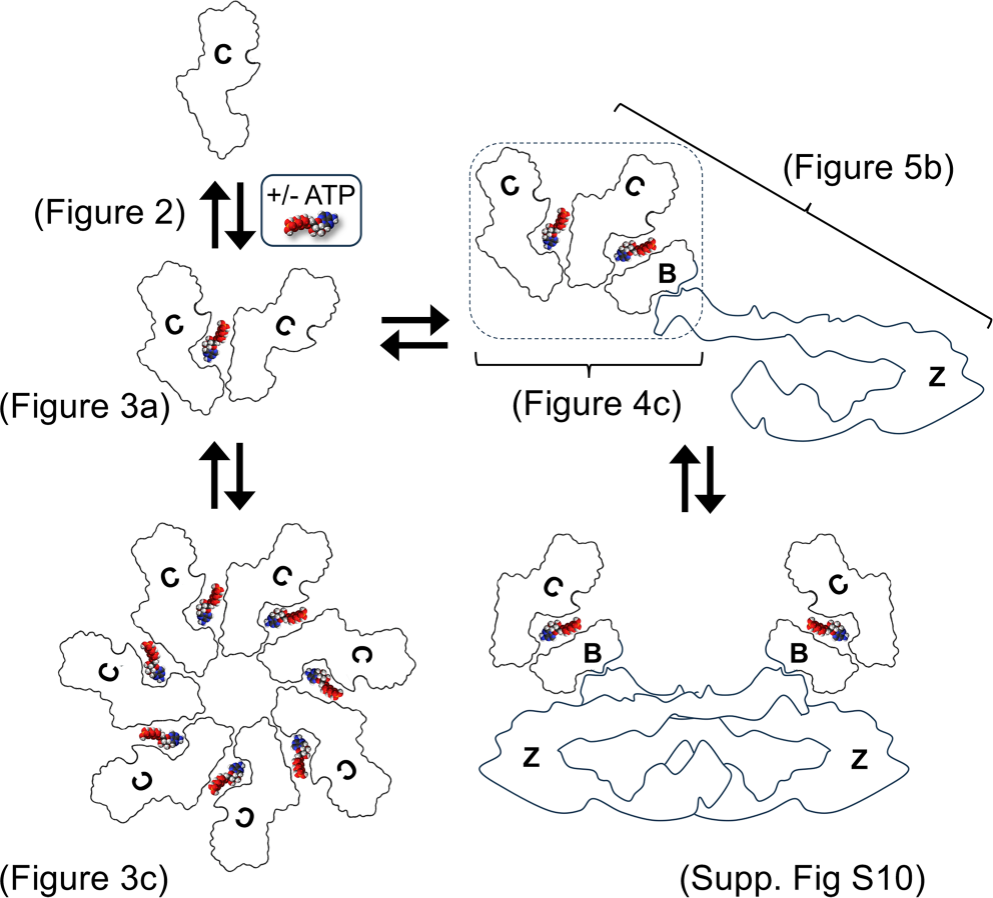
BrxC forms multiple assemblies. The schematic depicts the various structures formed by BrxC, based on findings in the indicated figures. For clarity, only BrxC’s N-terminal domain is represented.

We and others favor a model in which overlapping subsets of BREX proteins assemble into distinct complexes responsible for either host chromosome methylation or phage restriction [18, 21, 26, 39], with regulatory mechanisms coordinating dynamic transitions between these functional states. Here we observe that the BREX^Aci^ proteins BrxB, BrxC, PglZ, and PglX form a complex (“B:C:Z:X”) when co-expressed in *E. coli* (Supplementary Fig. S9); analogous complexes have also been reported in the *E. coli* and *Salmonella* type 1 BREX systems [21, 22]. Given the recently demonstrated nuclease activity of PglZ [23], we envision that phage infection induces remodeling of a methylation-specific complex into a restriction complex, thereby activating PglZ nuclease activity and potentially other BREX factors. The roles of BrxA and BrxL, which are required for restriction in some systems but dispensable in others [18, 26, 27] remains unresolved. Our study highlights the formation of B:C:Z complexes that may serve as a “base” for B:C:Z:X assemblies; and a potential central role for BrxC in (i) potentiating methylation and restriction and (ii) serving as a key point of regulation controlling the transition between these activities. The various structural states described in this study can be summarized as depicted in Fig. 7.

BrxC can self-assemble into at least two distinct structural states (Figs 3a, c, and 7). First, it forms a homodimer anchored by a tight C-terminal dimerization domain that creates a stable hinge, while its N-terminal AAA+ ATPase domains undergo reversible, ATP-regulated dimerization in an asymmetric “back-to-face” configuration (akin to the packing of spoons in a drawer), with key contacts formed between residues from one BrxC subunit and the ATP-binding pocket of its partner (Fig. 3a). A second structural state consists of higher order heptameric assemblies—observed with BrxC homologs from two different species—containing a central pore compatible with DNA binding (Fig. 3c). It is notable that we do not observe higher order assemblies when purified BrxC^Aci^ dimers are incubated only with ATP (Fig. 2), and only low-level tetramer formation with full-length BrxC^Eferg^ constructs (Supplemenatry Fig. S5), despite the geometries of BrxC’s AAA+ domains appearing compatible with the formation of AlphaFold3-generated heptameric complexes (Fig. 3c, bottom). The data are consistent with DNA facilitating BrxC oligomerization, which may include intermediates and higher order assemblies beyond the sparse heptameric particles we observe by negative stain EM (e.g. Fig. 3d). How and when these additional oligomer assemblies form, and their role in BREX activity, awaits further study.

We further demonstrate that BrxC associates with the stable PglZ:BrxB dimer through interactions centered on BrxC’s ATPase domain and BrxB’s inactivated AAA+ domain and can assemble into C_2_:B:Z: or C_2_:(B:Z)_2_ stoichiometries (Figs 4, 5 , and 7). In these configurations, BrxC forms a stable dimer mediated by its C-terminal domains, and its N-terminal AAA+ domains are accessible for binding BrxB. Combined, these results show that B:C:Z form a core complex that may assume various stoichiometries and suggest two structural roles for BrxB: (i) regulating higher-order BrxC oligomerization through its interaction with BrxC’s AAA+ domain, and (ii) bridging BrxC and PglZ within B:C:Z complexes.

In addition to demonstrating the formation of B:C:Z assemblages, we show that BrxB:PglZ regulates two activities of BrxC. First, BrxB:PglZ increases the ATPase activity of BrxC’s N-terminal ATPase domain, as shown when incubated with a BrxC_1–551_ construct that uncouples BrxC dimerization from ATPase activity (Fig. 5c). BrxB’s boost to BrxC’s ATPase activity may perhaps be due to facilitating ADP release, since BrxB^Eferg^ increases both BrxC^Eferg^_1–551_ and BrxC^Eferg^(E268Q)_1–551_ activity (Supplementary Fig. S12a). Second, BrxB:PglZ augments BrxC association with DNA (Fig. 1h). We also show that BrxA both binds BrxC and inhibits BrxC association with DNA (Fig. 1h). These findings indicate that BrxA and BrxB—recently shown to be universally present across all BREX subtypes [17]—may play important regulatory roles through their interactions with BrxC.

Genetic data indicate that the BrxB:BrxC:PglZ:PglX (B:C:Z:X) module is required for both the restriction and modification functions of the BREX system [18, 26, 27]. Consistent with this, mutations in the M.StyLT7II_BREX system have been identified within the C-terminal regions of PglX [40] and BrxC [38] that uncouple BREX-mediated methylation and restriction activities, likely by disrupting interactions with other BREX factors. Here, we show that a BrxB^Aci^(R82E) mutant that disrupts the BrxC-BrxB interface—demonstrated *in vitro* by both loss of binding and failure to augment BrxC^Aci^_1–551_ ATPase activity—also uncouples these activities, remaining largely competent for methylation but deficient in restriction. Together, these results provide functional evidence supporting a role for BrxB in regulating the coordination between BREX methylation and restriction activities through its interaction with BrxC.

Certain functional domains and enzymatic activities are repeatedly found across a wide range of bacterial defense systems, in accordance with the “guns for hire” paradigm in which enzymes have shuttled between mobile genetic elements (MGEs) and defense systems throughout microbial evolution [41, 42]. BREX systems have a high diversity of protein domains and activities, but are also notable for repeatedly using certain domains—for example the iSTAND and wHTH domains [17]. A recent bioinformatic analysis identified a family of BrxC homologs (DUF499) that retain strong N-terminal homology with canonical BREX BrxC proteins (DUF6079) but differ in their C-terminal regions [17]. The DUF499 BrxC variants co-occur with a PglX methyltransferase homolog and a helicase, and the locus invariably encodes an associated endonuclease. This three-gene configuration likely constitutes a bacterial defense system and has been termed a BREX-related (“BR”) system [17]. Of the three recognized BR subtypes, the type 3 subsystem lacks an endonuclease and instead encodes a minimal PglZ phosphoesterase domain together with a BrxB homolog—strongly supporting a functional role for PglZ’s DNA cleavage activity and the B:Z modules in BREX systems. BrxC homologs are also present in Dnd and Ssp systems [17, 43], highlighting BrxC as a conserved component across multiple bacterial defense systems. This prevalence is further highlighted by the phage-encoded BREX inhibitor OrbA that has been shown to inhibit BREX function by specifically targeting BrxC [44], which provides evidence for a central role of BrxC in mediating defense activity.

Our presented structures and functional data into the activity of conserved BrxC proteins provide another level of detail to the complex and elusive BREX mechanism. The increased identification of BREX-like functions across multiple defenses and the generalized nature of our findings will provide future opportunities to develop a fuller overview of BREX activity.

## Supplementary Material

gkag651_Supplemental_Files

## Data Availability

The crystal structure of BrxC^Aci^_1–551_:BrxB ^Aci^:PglZ ^Aci^_1–98_ has been deposited in the RCSB database (PDB DOI https://doi.org/10.2210/pdb9zll/pdb). The following cryo-EM models and corresponding maps have been deposited in the PDB and EMBD databases and will be publicly released April 8: BrxC ^Aci^(E269) dimer (PDB DOI https://doi.org/10.2210/pdb9zdx/pdb / EMD-74076), BrxC^Aci^_1–551_-BrxB^Aci^ fusion:PglZ^Aci^ dimer (PDB DOI https://doi.org/10.2210/pdb9zn5/pdb / EMD-74435), and BrxC^Aci^_1–551_-BrxB^Aci^ fusion:PglZ^Aci^ monomer (EMD-74400). Any other raw data has been deposited and made publically available at https://dataverse.harvard.edu/dataverse/BrxC/.
