# Supplementary material for: Multiple forms of protein–protein and DNA binding are exhibited by BrxC from the BREX phage restriction system
Source: Nucleic Acids Res. 2026 Jul 2;54(12):gkag651. doi: 10.1093/nar/gkag651 (PMC13326643; doi:10.1093/nar/gkag651)
Supplement: gkag651_Supplemental_Files [file gkag651_supplemental_files.zip › Supplementary_TableS1-S3-resubmit.pdf]

SUPPLEMTARY TABLE S1. PLASMIDS USED IN THIS STUDY

Acin BREX Constructs	Name	Vector	Construct	Purification	Tag/Terminus	Protease	Induction: Auto/IPTG	Antibiotic
	SII-TEV-BrxC FL WT	pET15b	BrxC FL WT	Streptactin	N-terminal	TEV	Auto	Amp
	SII-TEV-BrxC(E269Q) FL	pET15b	BrxC(E269Q)	Streptactin	N-terminal	TEV	Auto	Amp
	SII-TEV BrxC(D22C) FL	pET15b	BrxC(D22C)	Streptactin	N-terminal	TEV	Auto	Amp
	BrxC(1-1146)-Thr-SII	pET15b	BrxC(1-1147) WT	Streptactin	C-Terminal	Thrombin (not used)	IPTG	Amp
	BrxC(1-1146)E269Q-Thr-SII	pET15b	BrxC(1-1147)E269Q	Streptactin	C-Terminal	Thrombin (not used)	IPTG	Amp
	BrxC(1-551)-TEV-SII WT	pET15b	BrxC(1-551) WT	Streptactin	C-Terminal	TEV	IPTG	Amp
	BrxC(1-551)E269Q-TEV-SII	pET15b	BrxC(1-551)E269Q	Streptactin	C-Terminal	TEV	IPTG	Amp
	BrxC(1-551)R330E-TEV-SII	pET15b	BrxC(1-551)R330E	Streptactin	C-Terminal	TEV	IPTG	Amp
	PglZ-Thr-SII	pET15b	PglZ WT	Streptactin	C-Terminal	Thrombin	IPTG	Amp
	PglZ-Thr-SII	pET24d	PglZ WT	Streptactin	C-Terminal	Thrombin	IPTG	Kan
	PglZ(1-98)-Thr-SII	pET15b	PglZ(1-98)	Streptactin	C-Terminal	Thrombin	IPTG	Amp
	BrxB	pET24d	BrxB WT	Copurified	N/A	N/A	IPTG	Kan
	BrxB(R82E)	pET24d	BrxB(R82E)	Copurified	N/A	N/A	IPTG	Kan
	BrxB(S152C)	pET24d	BrxB(S152C)	Copurified	N/A	N/A	IPTG	Kan
	SII-TEV-PglX	pET15b	PglX WT	Streptactin	N-terminal	TEV	Auto	Amp
	BrxA-Thr-SII	pET15b	BrxA WT	Streptactin	C-Terminal	Thrombin	IPTG	Amp
	BrxC	pET24d	BrxC No Tag	Copurified	N/A	N/A	IPTG	Kan
	BrxB + BrxC	pET24d	BrxB + BrxC WT	Copurified	N/A	N/A	IPTG	Kan
	BrxB + BrxC(E269Q)	pET24d	BrxB + BrxC(E269Q)	Copurified	N/A	N/A	IPTG	Kan
	BrxB + BrxC(1-551)	pET24d	BrxB + BrxC(1-551)	Copurified	N/A	N/A	IPTG	Kan
	BrxA + BrxB + BrxC(E269Q)	pET24d	BrxA + BrxB + BrxC WT	Copurified	N/A	N/A	IPTG	Kan
	PglX + PglZ-SII	pET15b	PglX + PglZ-SII	Sreptactin	C-Terminal	Not Used	IPTG	Amp
	SII-TEV-PglZ + PglZ	pET15b	PglX + PglZ	Streptactin	N-Terminal	Not Used	IPTG	Amp
	H6-BrxC(1-553)-BrxB Fusion	pET15b	rxC(1-553)-BrxB Fusio	Co-purified	His6 - N-Termina	N/A	IPTG	Amp
	pACYC BREX WT	pACYC	WT BREX	N/A	N/A	N/A	N/A	Chlor
	pACYC BREX **Δ**BrxC	pACYC	BREX del.BrxC	N/A	N/A	N/A	N/A	Chlor
	pACYC BREX **Δ**BrxB	pACYC	BREX del.BrxB	N/A	N/A	N/A	N/A	Chlor
	pACYC BREX BrxC(E269Q)	pACYC	BREX + BrxC(E269Q)	N/A	N/A	N/A	N/A	Chlor
	pACYC BREX BrxC(R330E)	pACYC	BREX + BrxC(R330E)	N/A	N/A	N/A	N/A	Chlor
	pACYC BREX BrxC(1-1146) WT	pACYC	BREX + BrxC(1-1146)	N/A	N/A	N/A	N/A	Chlor
	pACYC BREX BrxC(1-1551) WT	pACYC	BREX + BrxC(1-551)	N/A	N/A	N/A	N/A	Chlor
	pACYC BREX BrxB(R82E)	pACYC	BREX + BrxB(R82E)	N/A	N/A	N/A	N/A	Chlor
	pACYC BREX BrxB(R185E)	pACYC	BREX + BrxB(R185E)	N/A	N/A	N/A	N/A	Chlor
**E. ferg BREX Constructs**	pTRB791	pSAT1-LIC	BrxC	IMAC	-SUMO - N-Term	hSENP2	Auto	Amp/Chlor
	pTRB792	pSAT1-LIC	BrxC(E268Q)	IMAC	-SUMO - N-Term	hSENP2	Auto	Amp/Chlor
	pTRB789	pSAT1-LIC	BrxC(1-551)	IMAC/AEC	-SUMO - N-Term	hSENP2	IPTG	Amp/Tet
	pTRB790	pSAT1-LIC	BrxC(1-551)E268Q	IMAC/AEC	-SUMO - N-Term	hSENP2	IPTG	Amp/Tet
	pTRB449	pSAT1-LIC	PglZ	IMAC/AEC	-SUMO - N-Term	hSENP2	IPTG	Amp
	pTRB444	pSAT1-LIC	BrxB	IMAC/AEC	-SUMO - N-Term	hSENP2	IPTG	Amp
	pBrxXL	pBrxXL	WT BREX	N/A	N/A	N/A	N/A	Chlor
	pBrxXL-**Δ**PglX	pBrxXL	BREX del.PglX	N/A	N/A	N/A	N/A	Chlor
	pBrxXL-**Δ**BrxU	pBrxXL	BREX del.BrxU	N/A	N/A	N/A	N/A	Chlor
	pBrxXL-**Δ**BrxU**Δ**PglX	pBrxXL	BREX del.BrxU/PglX	N/A	N/A	N/A	N/A	Chlor
	pBrxXL-**Δ**BrxC	pBrxXL	BREX del.BrxC	N/A	N/A	N/A	N/A	Chlor
	pBrxXL-BrxC(E268Q)	pBrxXL	BREX + BrxC(E268Q)	N/A	N/A	N/A	N/A	Chlor
	pBrxXL-BrxC(1-551)	pBrxXL	BREX + BrxC(1-551)	N/A	N/A	N/A	N/A	Chlor
**dsDNA Substrates**	100 bp PglX target centered	N/A	gcaggatcgaatcatggctggcgtcatgtaactacaggaatggtatg**GTAGAT**gatcgcatagattgatgcatacaatccgtttacggattcaggtaccg					

SUPPLEMENTARY TABLE S2. CryoEM Data

	**BrxC-E269Q**	**BrxCBZ dimer**	**BrxCBZ**
PDB/EMD-ID	9ZDX/EMD-74076	9ZN5/EMB-74435	EMD-74400
Data collection			
EM equipment	Glacios	Glacios	Glacios
Voltage (kV)	200	200	200
Detector	Gatan K3	Gatan K3	Gatan K3
Pixel size (Å/Pixel)	0.561	0.561	0.561
Electron dose (e-/Å^2)	50	50	50
Defocus range (um)			
Micrographs collected	4131	4012	3965
Micrographs used	3686	2509	3602
Reconstruction			
Software	cryoSPARC	cryoSPARC	cryoSPARC
# particles used	477,102	195,504	101,355
Symmetry	None	None	None
Resolution (Å)	2.95	7.02	4.46
Map sharpening B-factor (Å)	116.7	677.9	254.9
Refinement			
Software	phenix	N/A	N/A
Box			
Lengths (Å)	79.7, 96.5, 131.3	N/A	N/A
Angles (°)	90.0, 90.0, 90.0	N/A	N/A
Model composition			
Protein residues	1094	N/A	N/A
Nucleotide	0	N/A	N/A
Water	4	N/A	N/A
Ligands	4	N/A	N/A
MolProbity score	1.53	N/A	N/A
RMS Bonds (Å)	0.004	N/A	N/A
RMS Angle (°)	0.539	N/A	N/A
Ramachandran favored (%)	96.51	N/A	N/A
Ramachandran allowed (%)	3.49	N/A	N/A
Ramachandran outliers (%)	0	N/A	N/A

Supplementary Table S3. $BrxC_{1\text{-}551}$:BrxB:$PglZ_{1\text{-}98}$ crystallographic data and refinement

PDB code: 9ZLL

**Data Collection and Processing**		
Unit cell Dimensions	149.25 114.99 194.05 90.00 108.03 90.00	
Space Group	I2	
Average Mosaicity	0.17 °	
	Overall	Outer Shell
Low resolution limit	49.68	2.79
High resolution limit	2.74	2.74
Rmerge	0.085	0.711
Rpim	0.038	0.318
Total number of observations	555957	29943
Total number unique	81941	4446
Mean((I)/σ(I))	14.3	2.2
Mn(I) half-set correlation CC(1/2)	0.998	0.782
Completeness (%)	99.9	99.7
Multiplicity	6.8	6.7

**Model refinement**	
R_{work}	0.2195
R_{free}	0.2674
Mean Biso	69.1 Å^2
$RMSD_{bond}$	0.005 Å
$RMSD_{angles}$	0.98°
$RMSD_{dihedral}$	15.337°
Ramachandran distribution (Favored, allowed, disallowed)	92.85%, 5.54%, 1.61%
